# FGFR pathway inhibition enhances PD-1 blockade and modulates antitumor immunity in colorectal cancer models

**DOI:** 10.1186/s12885-026-17105-9

**Published:** 2026-10-13

**Authors:** Zhou Shang, Changqing Jing, Jiyuan Liu, Baolin Zhang, Marta Rúbies Bedós, Irineos Papakyriacou, Yumeng Mao, Yuezhi Chen

**Affiliations:** 1 Department of Gastrointestinal Surgery, Shandong Provincial Hospital Affiliated to Shandong First Medical University, Jinan, Shandong China; 2 Department of Gastrointestinal Surgery, Shandong Provincial Hospital, Shandong University, Jinan, Shandong China; 3 Science for Life Laboratory, Department of Immunology, Genetics and Pathology, Uppsala University, Uppsala, Sweden; 4 Department of Immunology, Genetics and Pathology, Uppsala University, Uppsala, Sweden

**Keywords:** FGFR signaling, Immune checkpoint blockade, MSS colorectal cancer, T cell exhaustion, Combination immunotherapy

## Abstract

Immune checkpoint blockade (ICB) has demonstrated limited efficacy in microsatellite stable (MSS) colorectal cancer (CRC). The fibroblast growth factor receptor (FGFR) signaling pathway contributes to tumor immune evasion, yet its role in CRC remains insufficiently understood. We investigated the immunomodulatory effects of FGFR inhibition in combination with PD-1 blockade in murine CRC models. In vitro co-culture systems were used to evaluate T cell activation upon FGFR3 blockade. RNA sequencing and bioinformatics analyses, including The Cancer Genome Atlas (TCGA) dataset evaluation, were conducted to elucidate molecular mechanisms and clinical relevance. Combined FGFR inhibition and PD-1 blockade significantly reduced T cell exhaustion and promoted macrophage repolarization toward an antitumor phenotype. Transcriptomic profiling revealed upregulation of immune-related pathways and activation of immune-stimulatory genes. In vitro, FGFR3-specific monoclonal antibody treatment enhanced IFNg release following PD-1 blockade. Moreover, analysis of TCGA data demonstrated that high *FGFR3* expression correlates with worse prognosis in CRC patients. Our findings suggest that FGFR pathway inhibition may enhance the efficacy of immune checkpoint blockade and provide a rationale for further investigation of FGFR-targeted combination strategies in MSS CRC.

**Figure:**
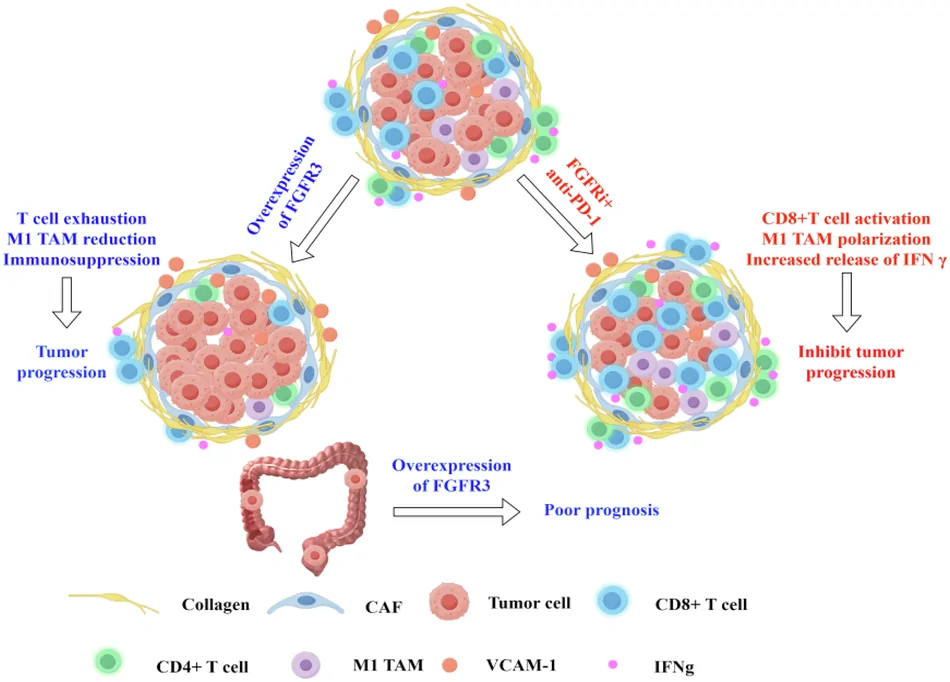

## Introduction

Immune checkpoint inhibitors (ICIs) have revolutionized the treatment of colorectal cancer (CRC) [1–3], particularly for patients with microsatellite instability-high (MSI-H) or deficient mismatch repair (dMMR) tumors [4, 5]. PD-1 blockade therapies, such as pembrolizumab and nivolumab, have shown durable responses and improved survival in these patients, leading to their accelerated U.S. Food and Drug Administration (FDA) approval for the treatment of CRC with MSI-H/dMMR [6–8]. Regrettably, the majority of CRC patients show limited response, if any, to monotherapy with ICIs, primarily due to the high incidence of mismatch repair-proficient (pMMR) and microsatellite-stable (MSS) tumors [9]. To address these resistance mechanisms and enhance PD-1 blockade efficacy, several combination therapies involving immune checkpoint blockade (ICB) and other anticancer agents, such as anti-EGFR, anti-VEGFR, or anti-FGFR drugs, are being actively investigated in clinical trials for patients with MSS/pMMR CRC [10, 11].

FGF/FGFR signaling plays a critical role in tumor progression, driving cell proliferation, survival, and angiogenesis, while also contributing to resistance against various cancer treatments [12, 13]. Notably, the combination of PD-1 blockade with FGFR inhibitors has already been approved for use in unresectable thyroid and hepatocellular carcinoma [14, 15]. However, despite these clinical advances, the immunomodulatory effects of inhibiting FGF/FGFR signaling and its impact on PD-1 blockade therapies, particularly in CRC, remain poorly understood.

In this study, we evaluated the combination of FGFR inhibitor and anti-PD-1 monoclonal antibody therapy in mouse models of CRC. The combination therapy demonstrated enhanced antitumor efficacy and significantly improved survival compared to monotherapy. Flow cytometry and immunofluorescence analyses revealed immune modulation within the tumor microenvironment. Additionally, RNA sequencing identified the upregulation of immune-related signaling pathways, providing mechanistic insights into the therapeutic synergy observed. Our in vitro experimental data, corroborated by TCGA analysis, revealed that *FGFR3* expression significantly contributes to immune regulation suppression and unfavorable clinical outcomes in CRC patients, thereby establishing its potential as a promising therapeutic target for CRC treatment.

## Methods

### Cell lines and culturing

CT26 and MC38 colon cancer cell lines were obtained from Saiye (Guangzhou) Biotechnology Co., Ltd. These cells were cultured in RPMI 1640 medium (KeyGEN BioTECH, KGM31800N-500) supplemented with 10% fetal bovine serum (OriCell, FBSSR-01021-50) and 100 U/ml penicillin-streptomycin (KeyGEN BioTECH, KGY0023) in a humidified incubator at 37°C with 5% CO₂.

The human colon cancer cell line HCT116 was kindly provided by Prof. Tobias Sjöblom (Rudbeck Laboratory, Uppsala University, Sweden). HCT116 cells were maintained in IMDM medium (Thermo Scientific) supplemented with 10% heat-inactivated fetal bovine serum and 1% penicillin-streptomycin (Thermo Scientific) at 37°C in a humidified atmosphere containing 5% CO₂. All cell lines were routinely tested for mycoplasma contamination using the MycoAlert kit (Lonza) and authenticated through DNA fingerprinting performed by Eurofins. The reagents used in the research institute are shown in Supplementary Table 1.

### In vivo CRC tumor models

To establish CRC tumor models, CT26 and MC38 colon cancer cells were digested into single-cell suspensions using trypsin (KeyGEN BioTECH, KGY0012) and diluted to a concentration of 5.0×10⁷ cells/mL in a 1:1 mixture of PBS (KeyGEN BioTECH, KGB5001) and Matrigel Matrix (082704, ABW&Mogengel). After a one-week acclimatization period, 6- to 8-week-old female BALB/c and C57BL/6 mice (*n* = 8) (Jinan; Xingkang Experimental Animal Co., Ltd.) were subcutaneously injected with 5×10⁶ CT26 or MC38 cells into the right flank. Tumor growth was monitored by measuring tumor volume with calipers, applying the formula: length × width² × 0.5. Mice were euthanized by inhalation of 100% carbon dioxide at a fill rate of 30% of the chamber volume per minute to minimize potential distress. This procedure was conducted in strict accordance with the American Veterinary Medical Association (AVMA) Guidelines for Euthanasia, and the study adhered rigorously to the ARRIVE guidelines.

### In vivo antitumor activity in the CRC tumor models

Mice were randomly assigned to different groups. Therapy began on day 0, when tumor volumes reached approximately 30–60 mm³ in BALB/c mice and 70–110 mm³ in C57BL/6 mice. Mice received intraperitoneal injections of 200 μg (10 mg/kg) anti-mouse PD-1 antibody (Bio X Cell, BE0146) or isotype control IgG (Bio X Cell) on days 0, 3, 6, and 9. AZD4547, a selective FGFR1–4 inhibitor (hereafter referred to as FGFRi), was used in the in vivo studies. FGFRi was administered orally at 10 mg/kg once daily from day 0 to day 14. Mouse weight and tumor volumes were measured every three days. Mice were euthanized when tumor volumes exceeded 2000 mm³, or if other humane endpoints were reached, including tumor ulceration or a 30% body weight loss compared to day 0. Survival duration (in days) was defined as the time from day 0 until euthanasia or death. Survival curves for each group were generated using GraphPad Prism software (version 10.4.1627,GraphPad Software, Inc., San Diego, CA, USA).

### Tumor rechallenge

Sixty days after the initiation of combination therapy, tumor-free C57BL/6 mice were rechallenged by subcutaneous injection of MC38 cells at double the original cell number into the contralateral flank, without administering further treatment. Age-matched naïve mice served as controls. Survival was monitored in both groups.

### Flow cytometry

In vitro experiments: HCT116 cells (1.5 × 10⁵) were seeded per well in 6-well plates and incubated overnight. The next day, cells were treated with or without 50 ng/mL recombinant human IFNg (rhIFNg, PeproTech) for 24 h. After incubation, cells were harvested by gentle scraping and stained with a panel of FACS antibodies (anti-PD-L1, 393606, Biolegend; HLA-ABC, 311410, Biolegend; IFNgRa, 308606, Biolegend; 0.2 ul per well) for flow cytometry analysis.

In vivo experiments: Spleens and tumors were harvested from experimental mice to isolate splenocytes and tumor-derived single cells. Tumor tissues were digested using a dissociation solution containing Type I collagenase, basic RPMI 1640 medium, and 4 µL DNase to obtain single-cell suspensions. After digestion, cells were washed, filtered, and treated to block non-specific binding. CD45^+^ cells and specific myeloid cells were then stained using antibodies described in the supplementary materials. Spleens were processed without digestion; they were directly minced, treated with red blood cell (RBC) lysis buffer, washed, and filtered. All samples were analyzed on a BD LSRFortessa X-20 flow cytometer, and data were processed using FlowJo software (version 10.8.1; FlowJo, LLC, Ashland, OR, USA).

### Immunofluorescence staining

Mice bearing with CRC tumors were treated with FGFRi and anti-PD-1 at specified doses for a defined duration. Mice received intraperitoneal injections of 200 μg (10 mg/kg) anti-mouse PD-1 antibody (Bio X Cell, BE0146) or isotype control IgG (Bio X Cell) on days 0, 3, 6, and 9. AZD4547, a selective FGFR1–4 inhibitor (hereafter referred to as FGFRi), was used in the in vivo studies. FGFRi was administered orally at 10 mg/kg once daily from day 0 to day 14. Two days after the final treatment, mice were euthanized, and tumor tissues were harvested. Samples were embedded in OCT compound (Sakura Finetek), frozen, and sectioned. Frozen sections were fixed in cold methanol. Immunofluorescence staining was performed using anti-CD8 (AB217344, Abcam, 1:200), anti-PD-1 (AB214421, Abcam, 1:1000), and anti-IFNg antibodies (A12450, Abclonal, 1:100) as primary antibodies, followed by secondary antibody (1:100) incubation with IgG. Hematoxylin and eosin (H&E) staining was used for nuclear counterstaining. Images of stained sections were captured and scanned for analysis. The areas positive for PD-1, CD8, and IFNg were quantified using SlideViewer software (version 2.9.0; 3DHISTECH, Budapest, Hungary).

### In vivo depletion of CD8^+^ T cells

Mice were intraperitoneally injected with 200 μg (10 mg/kg) anti-mouse CD8 antibody (Bio X Cell, BE0061) or Rat IgG2b isotype control (Bio X cell, BE0090) two days before treatment and 2, 6 and 10 days after treatment, respectively. Treatment commenced on day 0, when tumor volumes in BALB/c mice reached approximately 30–60 mm³. Mice received intraperitoneal injections of 200 μg (10 mg/kg) of anti-mouse PD-1 antibody (Bio X Cell, BE0146) or isotype control IgG (Bio X Cell, BE0089) on days 0, 3, 6, and 9. FGFRi (10 mg/kg) was administered orally daily from days 0 to 14. Mice were euthanized if tumor volumes exceeded 2000 mm³ or if humane endpoints, such as tumor ulceration or a 30% body weight loss relative to day 0 were reached.

### RNA-seq transcriptome analysis of tumor cells

Total RNA was extracted from CT26 colorectal cancer cells, and its quality was evaluated using the Agilent 2100 Bioanalyzer to confirm RNA integrity. Libraries were prepared from fragmented mRNA, quantified with a Qubit 2.0 Fluorometer, and diluted to a concentration of 1.5 ng/µL. The insert size of the libraries was verified using the Agilent 2100 Bioanalyzer.

Biological replicates were validated using the ggplot2 package in R (Version 3.0.3) to ensure reliability for differential gene analysis. Differentially expressed genes were clustered and visualized using ggplot2 and heatmap in R with Z-score row normalization. Total RNA was extracted from CT26 colorectal cancer cells, and its quality was evaluated using the Agilent 2100 Bioanalyzer to confirm RNA integrity. Libraries were prepared by Novogene and sequenced on the Illumina NovaSeq 6000 platform using a paired-end 150 bp (PE150) strategy. Raw sequencing reads were processed using fastp (version 0.19.7) for quality control, including the removal of adapter-containing reads, reads containing N bases, and low-quality reads. Clean reads were aligned to the mouse reference genome (GRCm38/mm10) using HISAT2 (version 2.0.5). Gene expression levels were quantified using featureCounts (version 1.5.0-p3), and differential expression analysis was performed using the DESeq2 R package (version 1.20.0), with genes showing |log2 (FoldChange)| > 1 and adjusted P-value < 0.05 considered differentially expressed. GO and KEGG enrichment analyses were performed using the clusterProfiler R package (version 3.8.1).

### Functional and enrichment analyses

Gene Ontology (GO) functional enrichment analysis was conducted using the clusterProfiler package in R. Enrichment analysis included all differentially expressed genes as well as separately upregulated and downregulated genes from the comparison groups. Gene Set Enrichment Analysis (GSEA) was performed with Java (Version 3.0), with all detected genes from both the combination therapy and anti-PD-1 monotherapy groups used as the input dataset. This analysis identified enriched pathways and GO terms that potentially contributed to the observed phenotypic differences.

### Co-expression and Venn diagram analysis

Co-expression analysis was conducted using the Venn Diagram package in R to identify overlapping genes between experimental groups. Intersecting gene sets were visualized using Venn diagrams, and shared gene IDs were highlighted to demonstrate common expression profiles across conditions.

### TCGA analysis

The Cancer Genome Atlas (TCGA) analysis was conducted using an online platform (https://www.home-for-researchers.com/). Data for colorectal cancer and immune-related correlations were sourced by downloading STAR-counts and corresponding clinical information from the TCGA database (https://portal.gdc.cancer.gov). Gene expression data were processed into Transcripts Per Million (TPM) format, followed by log2(TPM + 1) normalization. Only samples with both RNA-seq data and complete clinical annotations were included in subsequent analyses. Statistical analyses were conducted using R software (Version 4.0.3). Spearman’s correlation analysis was applied to assess monotonic associations between non-normally distributed variables. Statistical significance was defined as *p* < 0.05. For additional gene expression comparisons, data were retrieved from the publicly available Gene Expression Omnibus (GEO) database. Boxplots and corresponding statistical tests were also generated using R (Version 4.0.3), with *p* < 0.05 considered statistically significant.

### Human survival analysis

Kaplan–Meier survival analysis was conducted to assess the relationship between *FGFR3*

expression levels and clinical outcomes, including overall survival (OS) and relapse-free survival (RFS) in CRC patients. OS was defined as the time from initial diagnosis to death from any cause or last follow-up, whichever occurred first. RFS was defined as the time from curative surgery to disease recurrence or death from any cause, whichever occurred first. This analysis was performed using the Colon cancer mRNA Gene Chip dataset from the KMplot online tool (http://kmplot.com/analysis/). The survival curve was generated with the Kaplan-Meier Plotter (colon cancer) using gene 204380_s_at; after dividing the patients into groups, the follow-up threshold was set to “all” with “censor at threshold” checked, the user-selected probe set was used, and no restrictions were applied to subtypes, cohorts, or datasets (all). For expression grouping, to avoid missing correlations due to the use of a specific cutoff, all available cutoff values between the lower and upper quartiles of expression were evaluated for the selected gene, and the false discovery rate (FDR) using the Benjamini-Hochberg method was computed to correct for multiple hypothesis testing. The cutoff value with the highest significance (lowest FDR) was determined; in the case of multiple cutoff values with identical significance, the cutoff with the highest hazard ratio (HR) was selected for the final analysis. A *p*-value < 0.05 was considered statistically significant.

### Isolation of lymphocytes

Peripheral blood mononuclear cells (PBMCs) were isolated from buffy coats obtained from anonymous healthy donors at Uppsala University Hospital. Blood was layered over 15 mL of LymphoPrep solution in SepMate tubes (StemCell Technologies) and centrifuged at 1200 × g for 10 minutes. The resulting cells were collected, washed twice with 35 mL of PBS, and treated with RBC lysis buffer (BioLegend) at room temperature in the dark for 10 minutes. Lymphocytes were then enriched by depleting monocytes using the EasySep CD14^+^ selection kit (StemCell Technologies), according to the manufacturer’s instructions. The isolated lymphocytes were either used immediately or cryopreserved at ultra-low temperatures for future experiments.

### Tumor-immune co-culture system (TICS)

To establish the TICS, HCT116 cells were harvested and seeded in 96-well flat-bottom plates at 5,000 cells per well in 100 μL of cell culture medium, with or without lenvatinib (MedchemExpress) at varying concentrations or 5 μg/ml of anti-FGFR antibody (Bio-Techne), and incubated overnight at 37°C. The following day, lymphocytes isolated from healthy donors were labeled with Cell Trace Violet (CTV) dye (C34571, Thermo Fisher Scientific) in the dark for 10 min. After washing with PBS, lymphocytes were resuspended at 3 × 10⁶ cells/mL and added to the cancer cells in 100 μL of fresh culture medium, with or without 10 μg/mL of nivolumab (Bristol-Myers Squibb). After five days of co-culture, cytokine secretion (IFNg and granzyme B) was measured in the supernatants using ELISA (MabTech). Immune cell proliferation was subsequently analyzed by flow cytometry.

### Live imaging for cell proliferation

Untreated HCT116 cells (5 × 10³ cells per well) were seeded in 96-well flat-bottom plates with 100 μL of culture medium and incubated overnight. The following day, cells were treated with varying concentrations of lenvatinib. Cancer cell proliferation was monitored in real time using the Incucyte Zoom system (Sartorius). Cell confluence was measured at defined time intervals to calculate growth rates, and proliferation was tracked over a 5-day period.

### Statistical analysis

Flow cytometry data were analyzed using FlowJo version 10. All results were compiled and subjected to statistical analysis with GraphPad Prism version 9 or 10. For comparisons between experimental groups, one-way ANOVA was applied, with a significance threshold set at *p* < 0.05. Survival data were analyzed using GraphPad Prism 9 or 10, with statistical significance determined by the Log-rank (Mantel-Cox) test (*p* < 0.05). Comparisons between groups are denoted in the respective figure legends.

## Results

### FGFR inhibition synergizes with PD-1 blockade in tumor-bearing mice

**Fig. 1:**
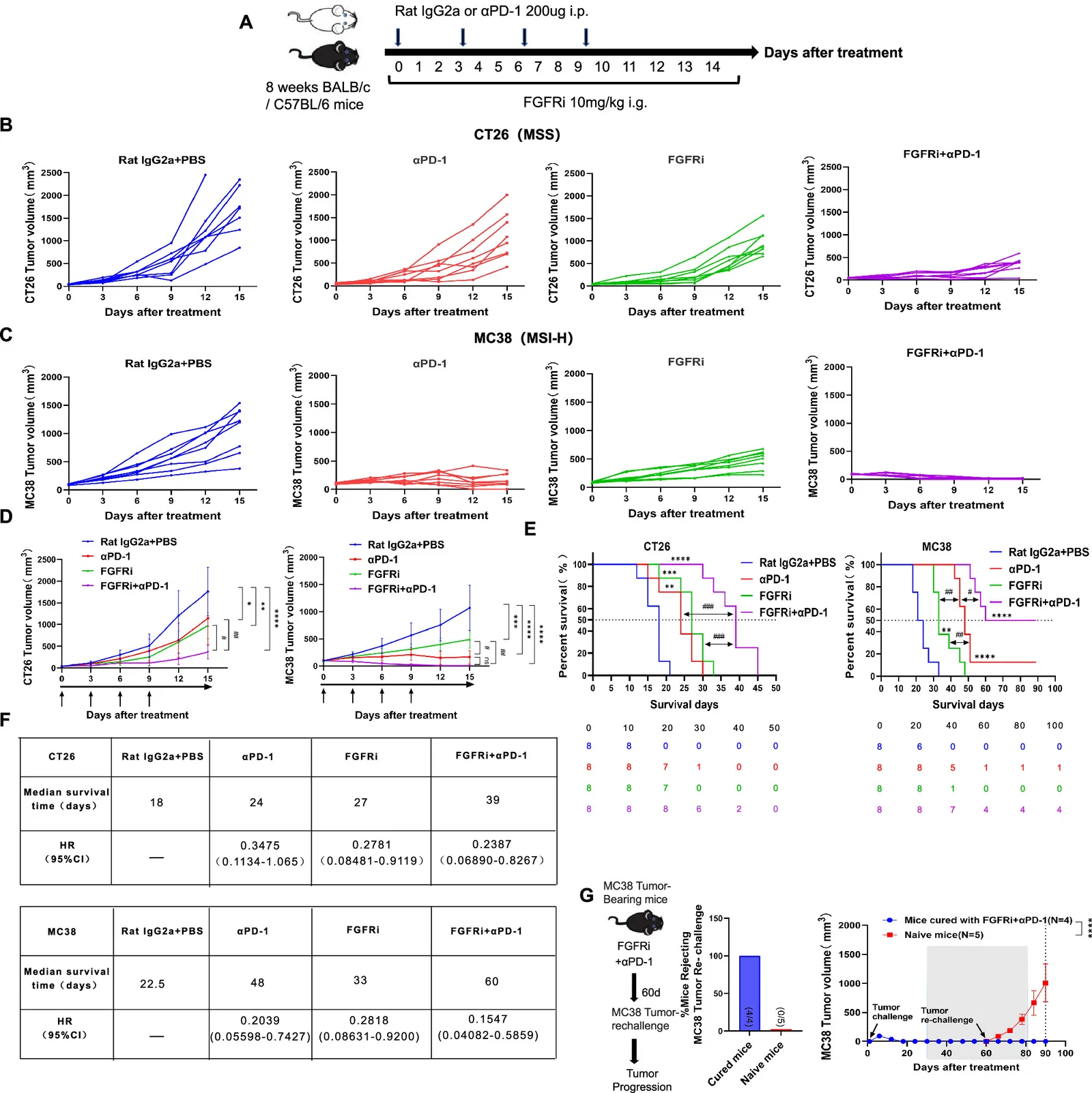
Evaluation of the antitumor activity of FGFRi combined with anti-PD-1 monoclonal antibody in CT26 (MSS) and MC38 (MSI-H) colorectal tumor models in BALB/c and C57BL/6 mice, respectively. **A**. Schematic diagram of the dosing schedule used in the CT26 and MC38 mouse tumor models. **B**. Individual tumor volume in the CT26 mouse tumor model. **C**. Individual tumor volume in the MC38 mouse tumor model. **D**. Average tumor volume in the CT26 and MC38 mouse tumor models. Black arrows indicate the treatment period. **E**. Kaplan-Meier survival curves for the mice. Survival time (in days) was defined as the period from Day 0 until death or humane euthanasia. **F**. Median survival time and hazard ratio (HR) with 95% confidence interval (CI) for each treatment group compared to the control group. Top panel: CT26 mouse tumor model; Bottom panel: MC38 mouse tumor model. **G**. Tumor reactivation in cured mice in the MC38 mouse tumor model. Left: schematic of the tumor rechallenge procedure in cured MC38 mice and corresponding survival outcomes. Right, average tumor volume in cured (n=4) and naive (n=5) mice in the MC38 tumor model.*, *p* < 0.05; **, *p* < 0.01; ***, *p* < 0.001; ****, *p* < 0.0001 vs. control group; #, *p* < 0.05; ##, *p* < 0.01; ###, *p* < 0.001 inter-group (Fig D, (one-way ANOVA with Tukey’s correction). Fig E, Simple survival analysis (Kaplan-Meier). Fig G, t tests (and nonparametric tests)); ns, not reached

We assessed the antitumor efficacy of FGFRi combined with anti-mouse PD-1 mAb in two syngeneic CRC mouse models: CT26 (MSS) and MC38 (MSI-H). Treatment began when tumor volumes reached 30-60 mm³ in BALB/c mice (CT26) or 70-110 mm³ in C57BL/6 mice (MC38). Anti-PD-1 mAb (200 µg/mouse) was administered intraperitoneally on days 0, 3, 6, and 9, while daily FGFRi (10 mg/kg) was administered orally for a duration of 14 days (Fig. 1A). In both models, monotherapy with either agent significantly inhibited tumor growth. In the CT26 model, FGFRi was more effective than anti-PD-1 mAb (Fig. 1B), whereas in the MC38 model, anti-PD-1 mAb demonstrated superior efficacy (Fig. 1C). The combination therapy exhibited significantly enhanced antitumor activity compared to either monotherapy (Fig. 1B, C).

In the CT26 model, although the combination therapy significantly suppressed tumor growth, no complete tumor regressions (tumor volume = 0) were observed (Fig. 1B). In contrast, the MC38 model demonstrated remarkable responses; one mouse treated with anti-PD-1 mAb alone achieved complete tumor regression by day 12, which persisted through the 90-day observation period. The combination therapy further improved outcomes, with complete tumor regression observed in 50% of the treated mice (4 of 8 mice; Fig. 1C). Average tumor volume changes in both models are presented in Fig. 1D. Importantly, no severe weight loss was observed in any treatment group compared to controls, underscoring the safety of the FGFRi and anti-PD-1 mAb combination (Supplementary Fig. S1A, B).

Survival curves for each treatment group in the CT26 and MC38 models are shown in Fig. 1E. Both FGFRi and anti-PD-1 mAb monotherapies significantly extended survival compared to the control group. However, the combination therapy demonstrated superior survival benefits in both models. In the CT26 model, median survival times were 18 days for the control group, 24 days for anti-PD-1 mAb, 27 days for FGFRi, and 39 days for the combination therapy. Similarly, in the MC38 model, median survival was 22.5 days for the control, 48 days for anti-PD-1 mAb, 33 days for FGFRi, and 60 days for the combination therapy (Fig. 1F).

Given that complete tumor regression was observed in some mice, we next evaluated the durability of the antitumor response by rechallenging cured mice with MC38 cells at 60 days post-treatment. All rechallenged mice completely rejected the tumors, while naïve control mice developed tumors as expected (Fig. 1G). These findings suggest that the combination therapy not only enhances survival but also induces robust, long-lasting immune protection

### Inhibition of FGFR enhances anti-tumor immunity upon PD-1 blockade in MSS CRC model

**Fig. 2:**
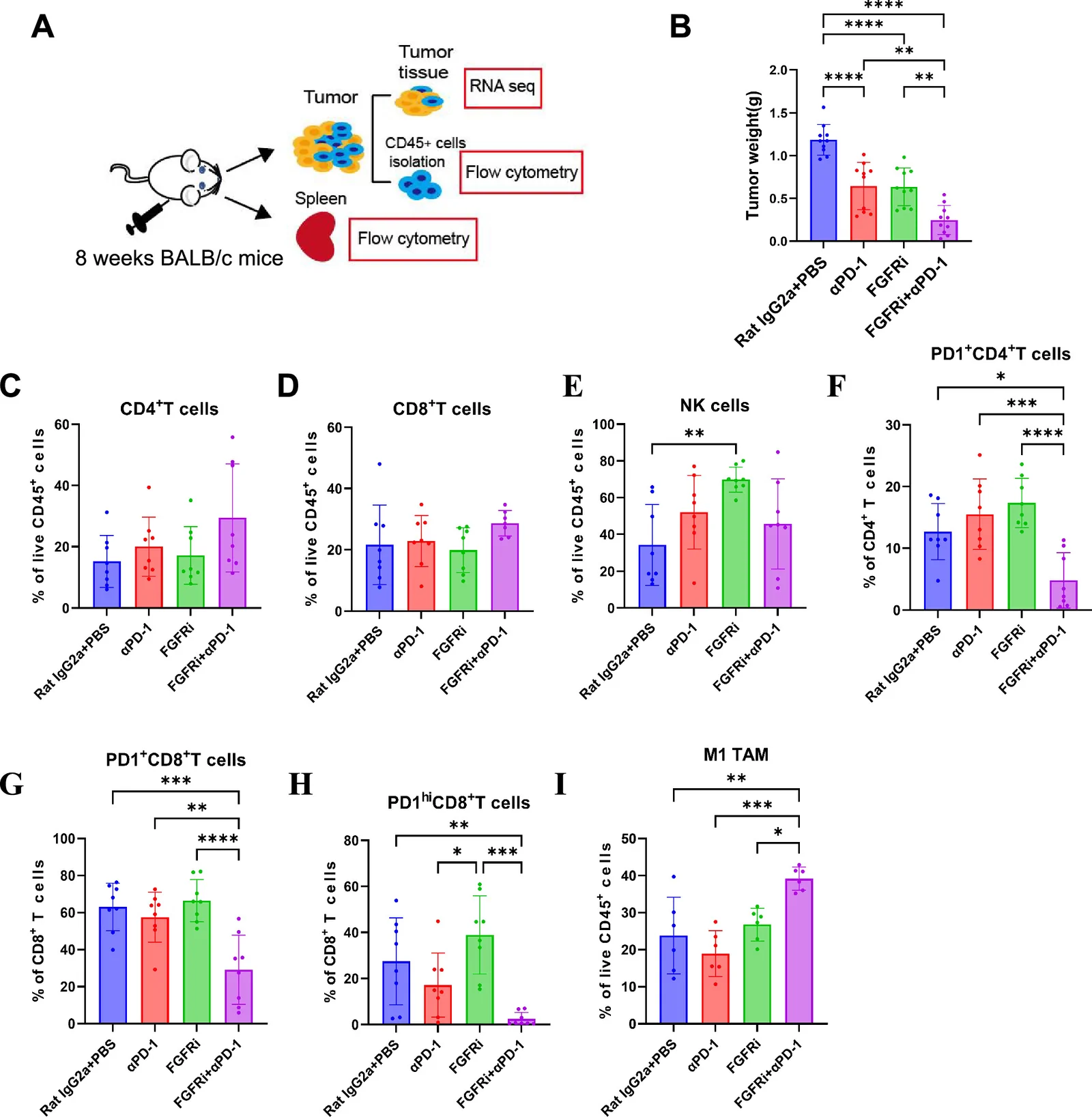
Inhibition of FGFR enhances anti-tumor immunity upon PD-1 blockade in MSS CRC model. **A**. Schematic workflow of flow cytometry and RNA-seq procedures following tumor and spleen harvest from treated mice. **B**. Mean tumor weight in each treatment group. **C-E**. Percentage of CD4+, CD8+ T cells and NK cells in CD45+ TILs. **F**. Percentage of PD1+ CD4+ T cells in CD4+ T cells. **G-H**. PD1+ CD8+ and PD1hi CD8+ T cells in CD8+ T cells. **I**. Percentage of M1 TAM in CD45+ TILs. *, *p* < 0.05; **, *p* < 0.01; ****, *p* < 0.0001 (one-way ANOVA with Tukey’s correction). n=8

To elucidate the immunomodulatory mechanisms underlying the antitumor effects of FGFRi in combination with anti-PD-1 mAb, we performed flow cytometric analysis of immune cell populations in CT26 tumors. Given the limited efficacy of PD-1 monotherapy in MSS tumors, we specifically aimed to determine whether inhibition of FGFR signaling could overcome immune resistance and enhance PD-1 responsiveness in MSS colorectal cancer (Fig. 2A). Tumor tissues were excised and weighed following treatment, revealing a significant reduction in tumor burden in the combination therapy group compared to either monotherapy (Fig. 2B).

CD45^+^ tumor infiltrating-lymphocytes (TILs) and myeloid-derived suppressor cells (MDSCs) isolated from tumors were evaluated based on the gating strategy shown in the Supplementary Fig. S2. Flow cytometry analysis revealed no significant changes in CD4^+^ and CD8^+^ T cell populations within the tumor microenvironment across treatment groups (Fig. 2C, D). However, the NK cell population was significantly increased in the FGFRi group compared to the control group (Fig. 2E). Notably, the combination therapy significantly reduced the percentages of PD-1^+^CD4^+^ and PD-1^+^CD8^+^ T cells (Fig. 2F, G) and abrogated the presence of PD-1^hi^CD8^+^ T cells (Fig. 2H). Additionally, the presence of M1 tumor-associated macrophages (TAMs) was significantly increased in the combination therapy group compared to the control and monotherapy groups (Fig. 2I), but there was no difference in the expression of M2 TAM among the treatment groups (Supplementary Fig. S3).

To evaluate the systemic immune response, we evaluated the immune composition in the spleens of the tumor-bearing mice using the gating strategy shown in Supplementary Fig. S4. We did not observe significant differences in CD4^+^ T cells, CD8^+^ T cells, NK cells, or PD-1^+^CD8^+^ T cells across treatment groups (Supplementary Fig. S5A-D). These findings suggest that the enhanced antitumor effects of combination therapy are associated with a substantial modulation of the tumor-infiltrating immune populations, including PD-1⁺CD4⁺ and PD-1⁺CD8⁺ T cells and M1 TAMs.

**Fig. 3:**
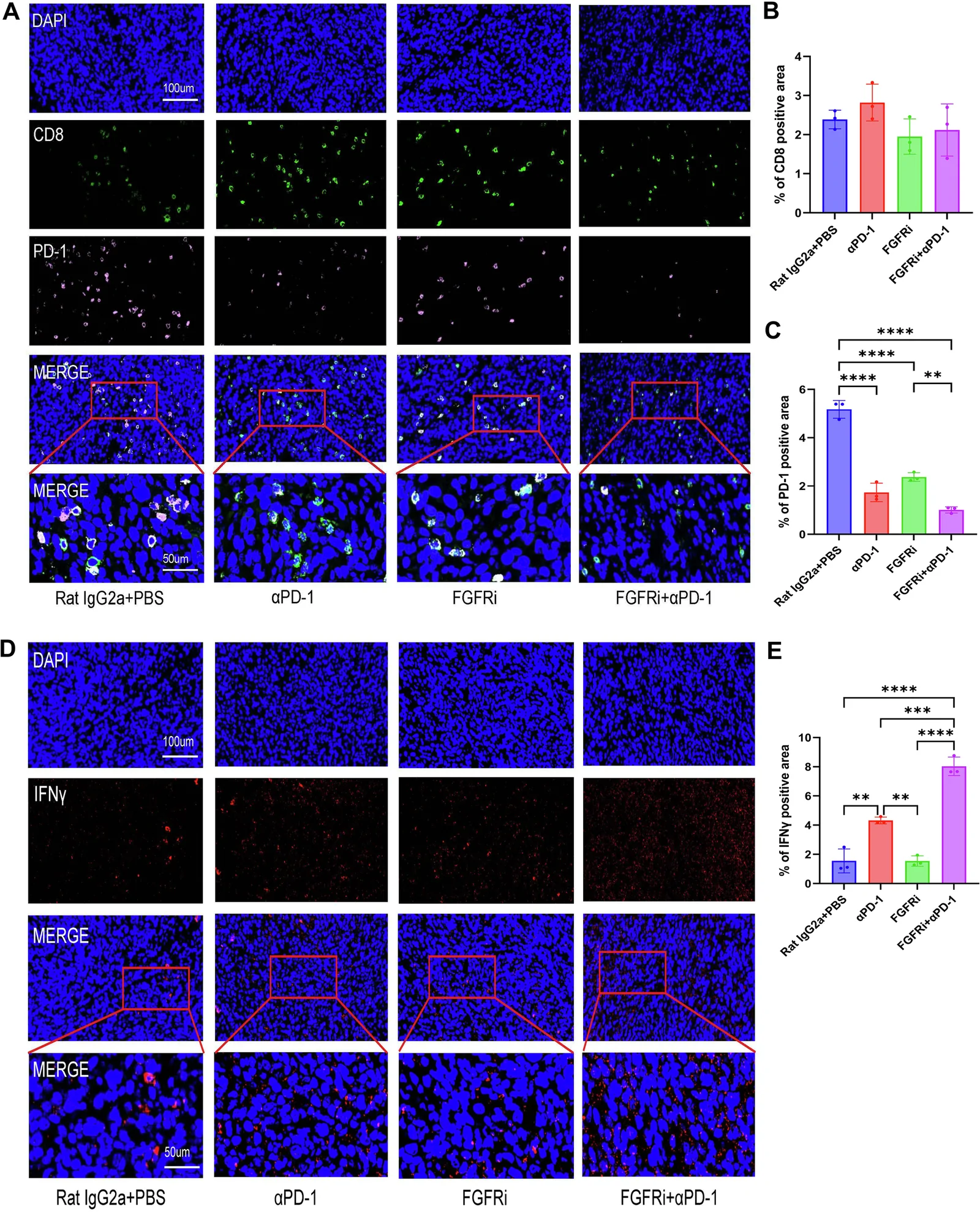
Immunofluorescence of CD8, PD1, and IFNg in CRC tumors. Mice bearing CT26 tumors were treated with FGFRi (10 mg/kg, Days 0–14, i.g.), anti-PD-1 (200 μg/mouse, Days 0, 3, 6, 9, i.p.), or their combination. **A**. Representative immunofluorescence images of CD8 and PD-1 in each treatment group. Scale bars: 50 μm and 100 μm. Data are presented as means ± SEM. **B**. Quantification of positive areas for CD8 in each treatment group. **C**. Quantification of positive areas for PD-1 in each treatment group. **D**. Representative immunofluorescence images of IFNg in each treatment group. Scale bars: 50 μm, 100 μm. Data are presented as mean ± SEM. **E**. Quantification of positive areas for IFNg in each treatment group. **, *p* < 0.01; ***, *p* < 0.001; ****, *p* < 0.0001 (one-way ANOVA with Tukey’s correction). (n=3)

To further validate our findings, immunofluorescence analysis was performed on tumor tissues from CT26 mice. Compared to the control group, CD8⁺ T cell expression showed no significant differences across treatment groups (Fig. 3A, B). However, PD-1 expression was reduced with anti-PD-1 monotherapy and further diminished with combination therapy (Fig. 3A, C). These findings are consistent with our flow cytometry results, suggesting that the enhanced antitumor effects of combination therapy may be driven by a reduction in exhausted T cells.

Additionally, we evaluated IFNg, a key cytokine involved in tumor cytotoxicity [16, 17]. Anti-PD-1 mAb monotherapy increased IFNg expression compared to the control group, while combination therapy further amplified this effect (Fig. 3D, E). These results suggest that the combination therapy not only reduces exhausted T cells but also enhances IFNg production, contributing to its superior antitumor efficacy.

### CD8⁺ T cell depletion abrogates the antitumor efficacy of combination therapy in the CT26 model

**Fig. 4:**
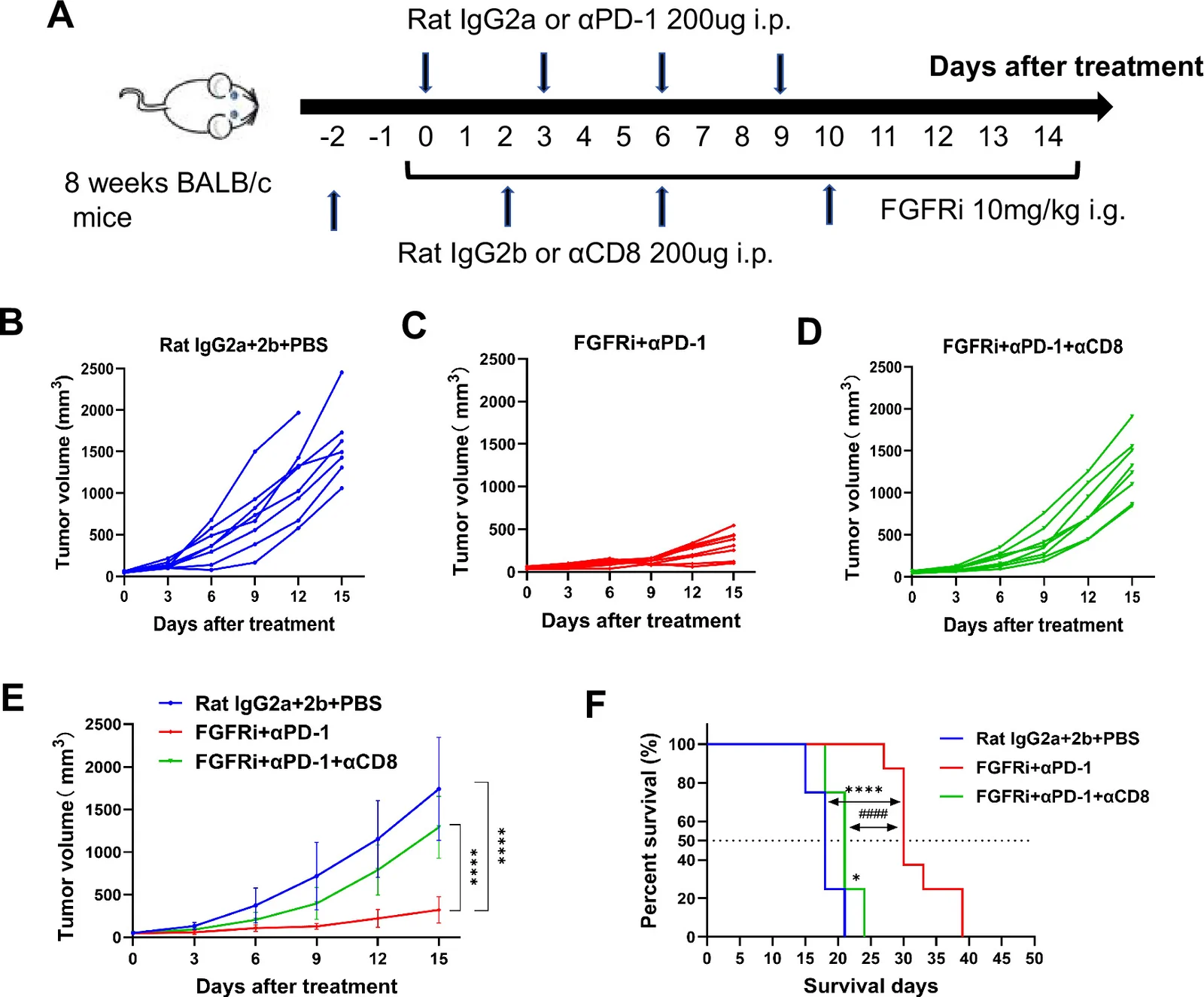
CD8⁺ T cell depletion abrogates the antitumor efficacy of combination therapy in the CT26 model. **A**. Schematic diagram of the CD8⁺ T cell depletion experiment protocol. **B-D**. Single mouse tumor volume in Rat IgG2 α+2b+PBS group. **C**. Single mouse tumor volume in FGFRi+anti-PD-1 group. **D**. Single mouse tumor volume in FGFRi+anti-PD-1+anti-CD8 group. **E**. Mean tumor volume for each treatment group. **F**. Kaplan–Meier survival curves of mice in each group. Survival time (in days) was defined as the period from Day 0 until death or humane euthanasia. *, *p* < 0.05; ****, *p* < 0.0001 vs. control group; ####, *p* < 0.001 inter-group (Fig E: one-way ANOVA with Tukey’s correction. Fig F: Kaplan–Meier survival analysis. n=8

To confirm that the anti-tumor effects of the combination therapy were dependent on CD8^+^ T cell activation, we compared the antitumor activity of combination therapy with and without CD8^+^ T cells in the CT26 CRC tumor-bearing mice by using anti-CD8 antibody (Fig. 4A). As shown in the gate strategy illustrated in Supplementary Fig. S6A, flow cytometry confirmed effective depletion of CD8^+^ T cells (Supplementary Fig. S6B, C). Antitumor efficacy analysis revealed that the combination therapy demonstrated significantly stronger tumor inhibition compared to the control group (Fig. 4B, C). However, this enhanced therapeutic effect was almost completely abolished when CD8^+^ T cells were depleted (Fig. 4D), highlighting their essential role in the therapeutic response. Average tumor volume changes in both models are presented in Fig. 4E. Additionally, the absence of CD8^+^ T cells markedly reduced the survival time of the mice (Fig. 4F), further underscoring the critical contribution of CD8^+^ T cells to the observed antitumor effects. No severe weight loss was observed in any treatment group compared to controls (Supplementary Fig. S7).

### Transcriptomics analysis reveals alterations of immune landscape in treated tumors

**Fig. 5:**
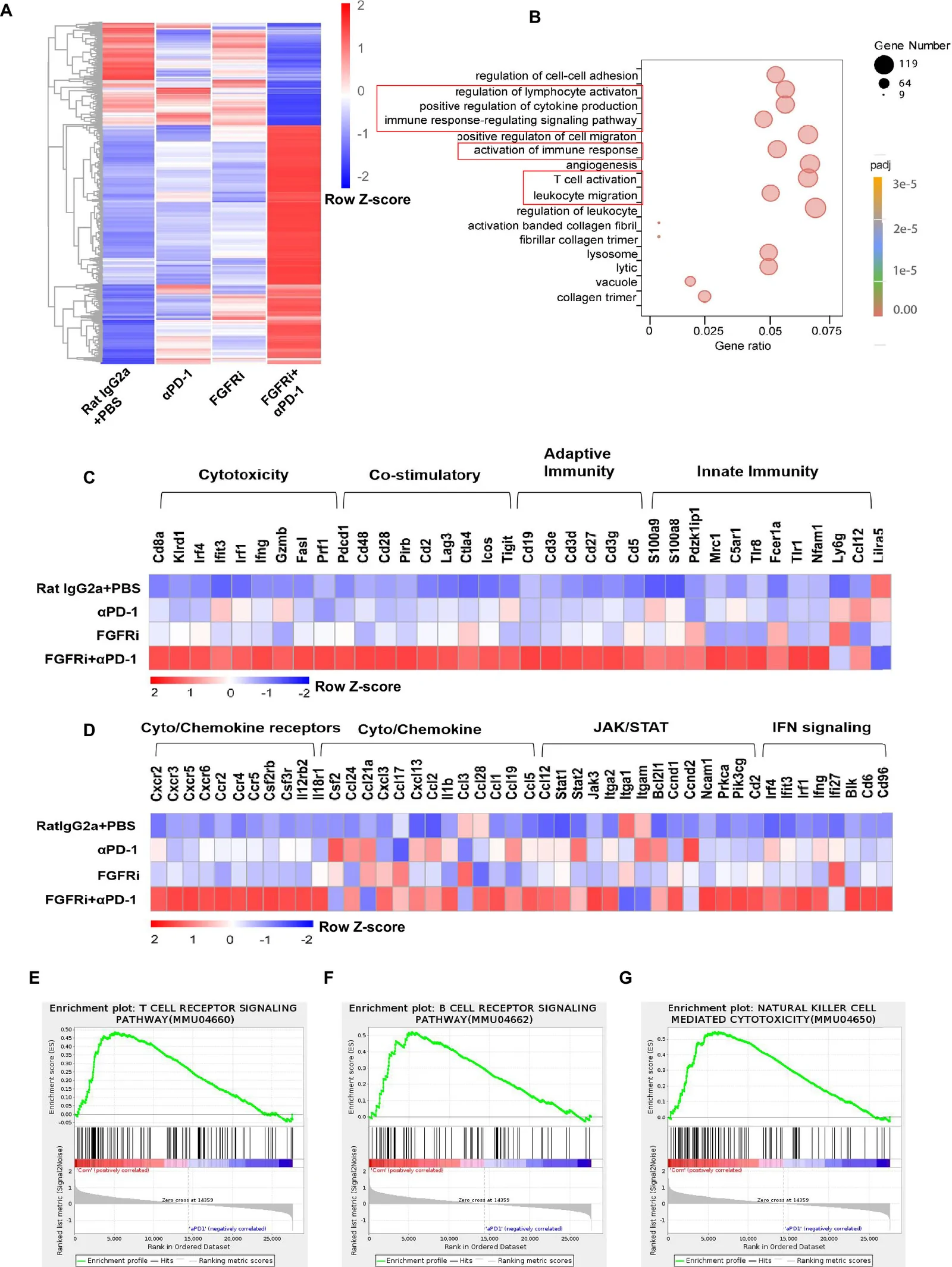
Transcriptomics analysis reveals alterations of immune landscape in treated tumors. RNA-seq was performed on tumor tissues from CT26-bearing mice in each treatment group. **A**. Heatmap showing clustering of differentially expressed genes across treatment groups. **B**. Gene ontology (GO) enrichment analysis of immune-related biological processes (combination vs. anti–PD-1). **C**. Expression levels of genes involved in cytotoxicity, immune costimulation, and adaptive/intrinsic immunity across treatment groups. **D**. Expression levels of genes related to chemokines and their receptors, JAK/STAT signaling, and interferon signaling pathways. **E-G**. Gene set enrichment analysis (GSEA) of pathways associated with T cell (**E**), B cell (**F**), and NK cell (**G**) signaling (combination vs. anti–PD-1)

To elucidate the molecular basis underlying the superior antitumor efficacy observed with combination therapy, we performed transcriptomic profiling of CT26 tumors using RNA sequencing. Given the central role of immune-regulatory genes in shaping antitumor responses [18], we focused our analysis on immune-related transcriptional changes. Notably, tumors from the combination group exhibited widespread differential gene expression compared to both monotherapy and control groups (Fig. 5A), indicating profound immune reprogramming. Gene enrichment analysis revealed that combination therapy upregulated gene expression in various immune-related biological processes, including T cell activation, cytokine production, and leukocyte migration (Fig. 5B). To further characterize the immune landscape in CT26 tumors, we categorized mRNA transcripts based on their biological functions. The analysis showed that combination therapy enhanced gene expression related to cytotoxicity, co-stimulatory molecules, and adaptive immunity (Fig. 5C). Moreover, combination therapy significantly upregulated genes involved in cytokine/chemokine signaling, JAK/STAT signaling and IFN signaling pathways (Fig. 5D), reinforcing the role of immune modulation in its antitumor efficacy.

Gene set enrichment analysis (GSEA) comparing the combination and anti-PD-1 monotherapy groups revealed enhanced expression in key immune signaling pathways, such as T cell receptor signaling, B cell receptor signaling and NK cell-mediated cytotoxicity (Fig. 5E–G). This increased activation of immune cell signaling pathways provides a mechanistic basis for the improved therapeutic outcomes observed with combination therapy, highlighting its potential as a strategic approach to enhance immune activation in colorectal cancer treatment.

### Lenvatinib enhances immune activation against human CRC cells in combination with nivolumab

To investigate the immunomodulatory activity of FGFRi in human cells, we utilized HCT116, a human colon cancer cell line from the FGFR genetic alteration panel, verified by American Type Culture Collection (ATCC) for FGFR pathway research and FGFR inhibitors anti–cancer drug discovery. Since IFNg pathway activation is linked to immunotherapy responses [19], we first assessed the responsiveness of HCT116 to recombinant human IFNg. Baseline analysis revealed that HCT116 cells exhibited high surface expression of HLA-ABC, PD-L1 and IFNGR1, but lacked HLA-DR expression. Upon IFNg stimulation, IFNGR expression was downregulated (Supplementary Fig. S8A), while PD-L1 expression was significantly upregulated (Supplementary Fig. S8B). However, HLA-ABC expression remained unchanged (Supplementary Fig. S8C). Next, we examined whether fibroblast growth factor 2 (FGF2), a mammalian FGF family ligand, or lenvatinib influenced immune-related marker expression in HCT116 cells. Neither treatment affected PD-L1, HLA-ABC, or IFNGR expression (Supplementary Fig. S8D), or the proliferation of HCT116 cells or the activation of human primary lymphocytes (Supplementary Fig. S8E, F).

**Fig. 6:**
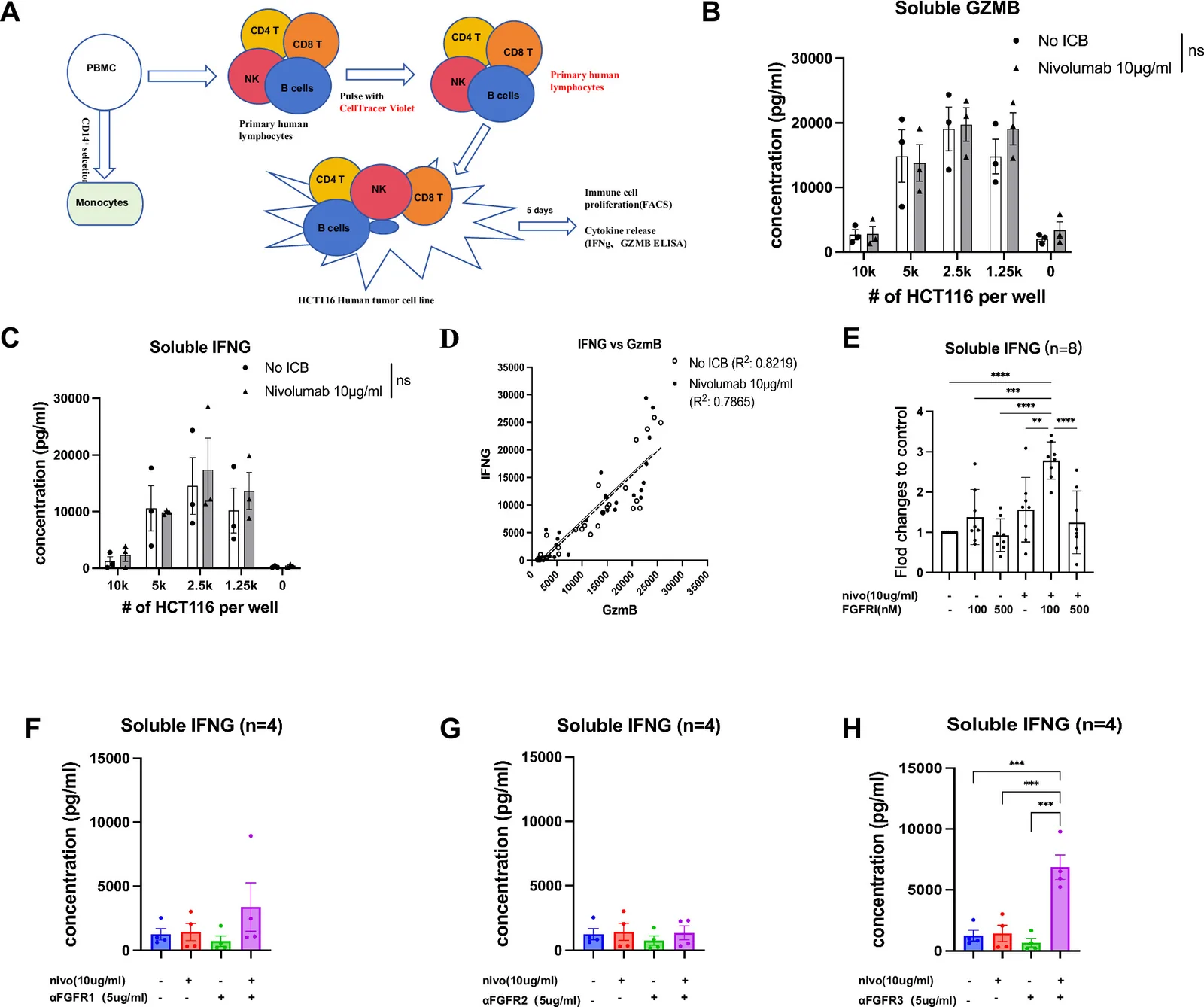
Lenvatinib enhances immune activation against human CRC cells in combination with nivolumab. **A**. Schematic diagram of tumor immune co-culture system (TICs). **B**. Expression of IFNg in cancer cells and lymphocytes at different ratios. **C**. Expression of GZMB in cancer cells and lymphocytes at different ratios. **D**. Correlation between IFNg and GZMB expression levels. **E**. Lenvatinib in combination with nivolumab significantly increased IFNg production. **F**. Blocking FGFR1 had no significant effect on the response to PD-1 blockade. **G**. Blocking FGFR2 had no significant effect on the response to PD-1 blockade. **H**. Blocking FGFR3 enhanced the response to anti-PD-1 blockade.**, *p* < 0.01; ***, *p* < 0.001; ****, *p* < 0.0001 vs. isotype (Data are presented as mean ± SEM. one-way ANOVA with Tukey’s correction)

To further explore the immunomodulatory effects of lenvatinib and PD-1 blockade, we established and optimized a tumor-immune co-culture system (TICS; Fig. 6A). This system enables the evaluation of both the response and resistance of clinically approved drugs targeting the PD-1/L1 axis in vitro [20, 21]. As shown in Fig. 6B and C, IFNg and granzyme B production from human lymphocytes depended on the cancer cell-to-lymphocyte ratio, with the 2.5 K ratio providing the most robust lymphocyte activation. Interestingly, nivolumab alone did not enhance the release of IFNg or granzyme B in the TICS (Fig. 6B, C). Additionally, IFNg and granzyme B levels were strongly correlated across donors in both the control (R² = 0.8219) and nivolumab-treated groups (R² = 0.7865; Fig. 6D). Given this correlation, subsequent experiments focused on IFNg as a key immunomodulatory marker. We then evaluated the combined effect of lenvatinib and nivolumab in the TICS model. As shown in Fig. 6E, the combination of lenvatinib (100 nM) and nivolumab significantly increased IFNg production. Since lenvatinib is a multi-target inhibitor with off-target characteristics [22], we repeated the experiment using monoclonal antibodies to block specific FGFR receptors. Our results demonstrated that FGFR3 blockade specifically enhanced the response to anti-PD-1 treatment (Fig. 6F-H), suggesting that FGFR3 inhibition plays a pivotal role in modulating tumor-immune interactions.

### High FGFR3 expression correlates with poor prognosis and immune suppression in CRC

**Fig. 7:**
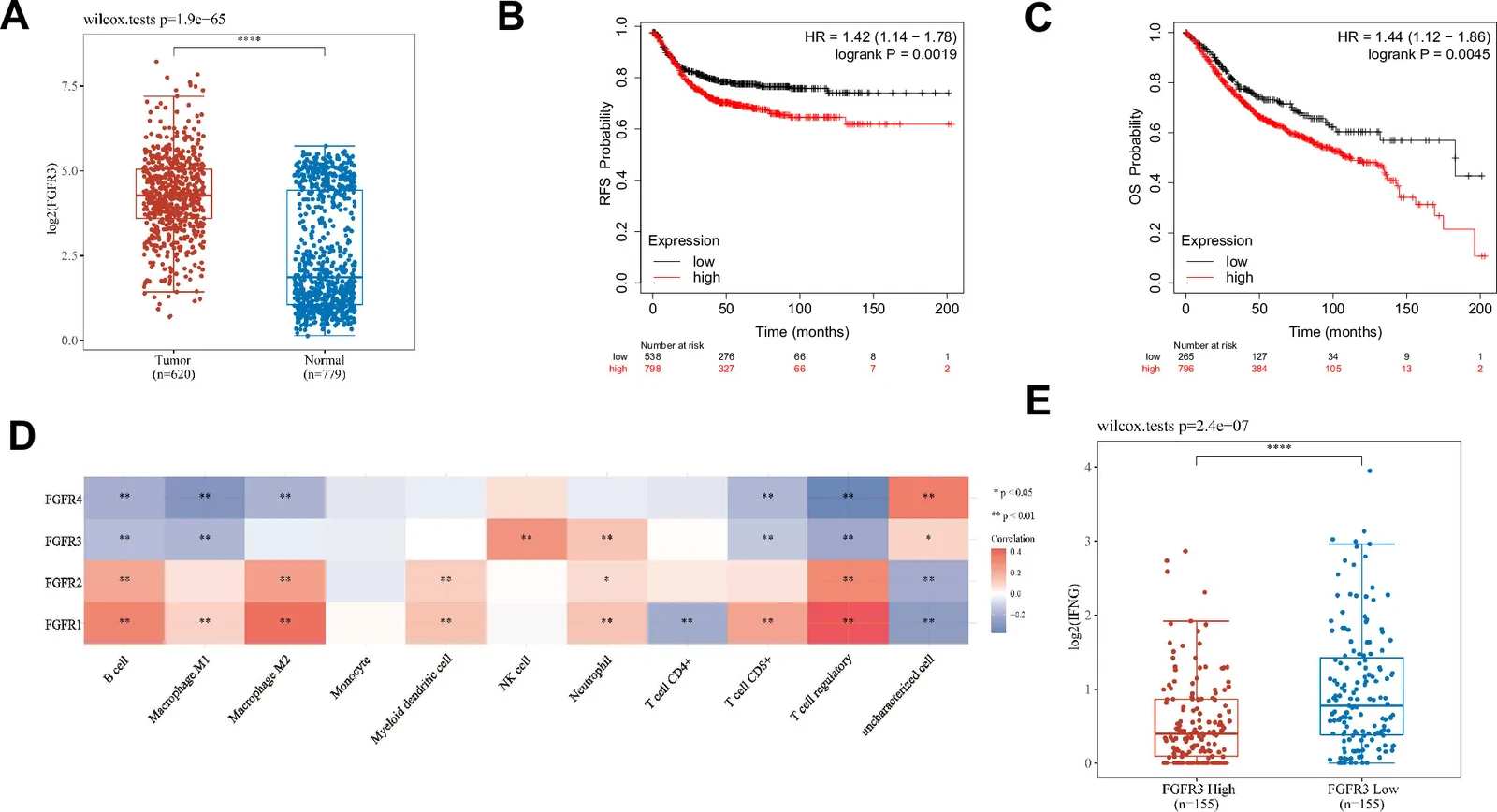
High FGFR3 expression correlates with poor prognosis and immune suppression in CRC patients. **A**. Box plot showing FGFR3 gene expression levels in CRC tumor tissues versus normal tissues. **B**. Relapse-free survival (RFS) of CRC patients stratified by FGFR3 expression levels. **C**. Overall survival (OS) of CRC patients stratified by FGFR3 expression levels. **D**. Correlation between FGFR3 expression and immune cell infiltration levels. **E**. Box plot showing the relationship between FGFR3 expression and IFN-γ expression in CRC tumor tissues. (*, *p* < 0.05; **, *p* < 0.01; ****, *p* < 0.0001. Wilcoxon test: FGFR3 low group vs. FGFR3 high group and normal group vs. Tumor group

Our study demonstrates that *FGFR3* exerts a significant immunosuppressive role in CRC. To further validate these findings, we investigated the association between *FGFR3* expression, immune regulation, and the prognostic outcomes in CRC patient datasets. Compared to normal colorectal tissue, *FGFR3* mRNA expression was significantly elevated in CRC tissue (Fig. 7A). Clinically, patients with high *FGFR3* expression exhibited worse prognosis, with significantly shorter recurrence-free survival (RFS) and overall survival (OS) compared to those with low *FGFR3* expression (Fig. 7B, C). Moreover, *FGFR3* expression showed a significant inverse correlation with key immune cell populations, including NK cells and CD8⁺ T cells (Fig. 7D), indicating its role in immune suppression. Additional analyses revealed that *FGFR3* expression significantly inhibits IFNg production (Fig. 7E), further supporting its association with an immune-suppressive tumor microenvironment. Collectively, these findings underscore *FGFR3*’s detrimental impact on immune regulation and patient survival, highlighting its potential as a promising immunotherapeutic target in CRC.

## Discussion

Although targeted therapies and immunotherapies have demonstrated some clinical potential [23], neither strategy has yielded consistent or durable responses when used as monotherapies in CRC, particularly in the majority of pMMR/MSS tumors [24]. To improve therapeutic outcomes, current research is increasingly focused on rationally designed combination strategies that integrate these approaches [25, 26]. We used the MC38 and CT26 syngeneic CRC mouse models, which differ in their genetic mutation profiles and microsatellite stability [27–29]. Our research indicated that anti-PD-1 monotherapy exhibited greater efficacy in the MC38 model, which has a higher mutational burden than the CT26 model. This result aligns with clinical observations of ICB monotherapy responses in MSI/dMMR CRC patients [30]. However, when compared to anti-PD-1 monotherapy, FGFR inhibition (10 mg/kg) as a monotherapy exerted robust anti-tumor effects in both models. In addition, regardless of the tumor model, the combination of FGFRi and anti-PD-1 significantly enhanced anti-tumor activity and markedly prolonged survival compared to either treatment alone. This durable suppression of tumor growth may be attributed to enhanced immune activation, suggesting that FGFR inhibition not only potentiates the efficacy of anti-PD-1 therapy but also sustains immune-mediated tumor control by modulating the tumor microenvironment.

Given that anti-PD-1 monotherapy only moderately inhibited tumor growth in the CT26 model, we performed an immune-related analysis to explore the mechanisms underlying the effective and sustained tumor suppression induced by FGFRi+anti-PD-1 combination therapy. In our investigation of the CT26 tumor model, we found that the combination therapy significantly reduced the proportions of PD-1^+^CD4^+^ and PD-1^+^CD8^+^ T cells compared to both the control and monotherapy groups. Notably, PD-1^hi^CD8^+^ T cells were almost undetectable in the combination therapy group. Since elevated PD-1 expression is a hallmark of T cell exhaustion [31, 32], these results suggest that combining FGFR inhibition with anti-PD-1 therapy reduces the prevalence of exhausted T cells within the tumor microenvironment (TME), thereby enhancing anti-tumor efficacy. However, the underlying molecular mechanisms driving this reversal of exhaustion remain to be fully elucidated. Our findings demonstrate that FGFRi monotherapy exerted robust antitumor effects in both syngeneic CRC models, while combining FGFRi with anti-PD-1 further suppressed tumor growth and markedly prolonged survival. Mechanistically, the combination therapy attenuated T cell exhaustion and promoted M1 macrophage polarization in the tumor microenvironment, with activation of JAK/STAT and IFN signaling pathways and enhanced IFNγ production. These results support FGFR inhibition combined with PD-1 blockade as a promising strategy for MSS CRC and provide a rationale for further clinical evaluation.

Interestingly, unlike previous studies [24, 33–35], our combination regimen did not increase the overall levels of CD4⁺ and CD8⁺ T cells in the tumor microenvironment. This discrepancy may be attributed to the timing of tumor collection for flow cytometric analysis. Tumors were harvested one day after the final FGFR inhibitor (FGFRi) administration (day 15), following euthanasia. This timing likely captured a transient phase of the immune response, during which CD4⁺ and CD8⁺ T cell populations might have expanded earlier but were no longer elevated at the time of analysis. This hypothesis was supported by subsequent CD8⁺ T cell depletion experiments. Additionally, recent research on liver cancer has demonstrated that tumor-resident immune subtypes, such as CD4⁺ regulatory T cells (Tregs) and KIR⁺CD8⁺ T cells, can influence immunotherapy efficacy [36]. In CRC, further investigation is needed to determine whether combination therapy affects the immune regulatory activity of these specific cell populations.

In addition to reducing exhausted T cells, our data revealed that FGFR inhibition and anti-PD-1 therapy together promotes sustained M1 macrophage polarization, a phenomenon observed only in the combination therapy group. This M1 polarization may be driven by increased IFNg release from activated T cells in the combination therapy group, as IFNg is a key cytokine for M1 induction [37, 38]. This hypothesis is supported by our immunofluorescence analysis. Previous studies have demonstrated that inhibiting FGF/FGFR signaling modulates macrophage programming and plays a key immunoregulatory role in the tumor microenvironment [39, 40]. Our findings align with these reports, highlighting FGFR inhibition as a crucial mechanism for enhancing the anti-tumor effects of anti-PD-1 therapy through macrophage regulation. Surprisingly, no significant differences in immune cell populations were found in the spleen across treatment groups, suggesting that the immunomodulatory effects of the combination therapy are primarily localized within the TME rather than affecting systemic immunity.

Our findings show that PD-1 blockade alone does not significantly alter the expression of genes related to cytotoxicity, adaptive immunity, or cytokines/chemokines. However, combination therapy markedly modulated the expression of immune-related genes, though the exact molecular mechanisms underlying this effect warrant further investigation. Notably, our data reveal a significant enrichment of genes associated with the JAK/STAT and IFN signaling pathways, which may explain the markedly higher expression of IFNg observed in the tumor microenvironment of the combination therapy group, as confirmed by immunofluorescence. Previous studies in renal cancer have shown that JAK/STAT pathway-related gene expression is significantly upregulated in response to FGFR inhibition combined with anti-PD-1 therapy, suggesting potential crosstalk between the FGFR and JAK/STAT pathways [41]. This interaction may represent a key mechanism underlying the enhanced antitumor response and warrants further investigation and validation.

Lenvatinib is an oral multi-target tyrosine kinase inhibitor that selectively inhibits FGFR1–4, VEGFR1–3, RET, and KIT [42]. It has been clinically approved for the treatment of thyroid cancer, hepatocellular carcinoma and renal cell carcinoma [43–46]. Our tumor immune co-culture system demonstrated that nivolumab alone did not enhance IFNg secretion in TICS, whereas the combination of lenvatinib and nivolumab significantly increased IFNg production. Given lenvatinib’s multi-target nature [22], we further investigated the specific FGFR subtype responsible for this effect. FGFR3-specific blockade showed the most pronounced effect among the tested FGFR subtypes in enhancing the response to PD-1 blockade in human CRC cells, suggesting that FGFR3 may contribute to immune regulation in this context. Previous studies in liver cancer have shown that lenvatinib can enhance the efficacy of PD-1 blockade by targeting FGFR4 [47]. Our findings provide additional evidence that FGFR signaling may participate in immune regulation in CRC. TCGA database analysis further confirmed that *FGFR3* overexpression correlates with heightened immune suppression and poorer prognosis in CRC. However, the specific contribution of FGFR3 to the in vivo therapeutic response remains to be determined. Further FGFR3-specific genetic and pharmacological studies are required to clarify its precise role in CRC immunotherapy.

Several limitations of this study should be acknowledged. First, AZD4547 used in the syngeneic mouse models inhibits multiple FGFR family members, and therefore the individual contribution of specific FGFR subtypes to the therapeutic response cannot be fully determined. Second, although FGFR3-specific blockade showed potential involvement in human CRC cells, further in vivo validation is required to establish its causal role in immune modulation. Third, while RNA sequencing identified alterations in immune-related pathways, additional immune populations, including NK cells and neutrophils, may also contribute to the response to FGFR pathway inhibition combined with PD-1 blockade and require further investigation. Body weight was monitored throughout the study as an indicator of treatment tolerability. Although a maximum 30% body weight loss was predefined as the humane endpoint, only one animal experienced body weight loss exceeding 20%, while all other animals remained below 20%. No significant differences in body weight were observed between treatment groups and controls, and the combination regimen did not result in additional weight loss compared with monotherapy. These findings suggest that FGFR pathway inhibition combined with anti-PD-1 was well tolerated under the conditions of this study, without apparent additional systemic toxicity.

## Conclusion

Our findings demonstrate that FGFR pathway inhibition enhances the antitumor activity of PD-1 blockade in colorectal cancer models, accompanied by reduced T cell exhaustion and increased antitumor immune activation. FGFR3-specific blockade further enhanced PD-1 blockade in human CRC cells, suggesting a potential role of FGFR3 in immune regulation. Further studies are needed to determine the specific contribution of individual FGFR subtypes, particularly FGFR3, to the in vivo response.

## Supplementary information

Supplementary Information

Supplementary Information

Supplementary Information

Supplementary Information

Supplementary Information

Supplementary Information

Supplementary Information

Supplementary Information

Supplementary Information

Supplementary Information

## Data Availability

The raw RNA-seq data generated in this study are available in the National Center for Biotechnology Information (NCBI) GEO database under accession number GSE347086 (https://www.ncbi.nlm.nih.gov/geo/query/acc.cgi?acc=GSE347086).
