# Supplementary material for: FGFR pathway inhibition enhances PD-1 blockade and modulates antitumor immunity in colorectal cancer models
Source: BMC Cancer. 2026 Oct 13;26:1227. doi: 10.1186/s12885-026-17105-9 (PMC13670699; doi:10.1186/s12885-026-17105-9)
Supplement: Supplementary file 1 [file 12885_2026_17105_MOESM10_ESM.pdf]

Colon cancer cell lines				
Name	Clone	Fluorochroe	Catalog number	Supplier
HCT116.WT	none	none	H1-0401	Rudbeck Laboratory
CT26.WT	none	none	M1-0101	Sai Ye
MC38.WT	none	none	M1-0401	Sai Ye
PBMCs	none	none	none	Uppsala University Hospital
In vitro antibodies and reagents				
Lenvatinib	none	none	HY-10981	MedchemExpress
Anti-FGFR1 antibody	none	none	MAB765	Bio-Techne
Anti-FGFR2 antibody	none	none	MAB6843	Bio-Techne
Anti-FGFR3 antibody	none	none	MAB7661	Bio-Techne
Nivolumab	none	none	none	Bristol-Myers Squibb
anti-PD-L1	none	none	393606	Biolegend
HLA-ABC	none	none	311410	Biolegend
IFNgRa	none	none	308606	Biolegend
In vivo antibodies and reagents				
Anti-PD-1	RMP1-14	none	BE0146	BioXCell
Anti-CD8a	2.43	none	BE0061	BioXCell
Rat IgG2b Isotype Control	LTF-2	none	BE0090	BioXCell
Rat IgG2a Isotype Control	2A3	none	BE0089	BioXCell
AZD4547	none	none	HY-13330	MedchemExpress
InVivoPure pH 7.0 Dilution Buffer	none	none	IP0070	BioXCell
Matrigel Matrix	none	none	082704	ABW&Mogengel
Cell antibodies used for flow cytometry				
Aqua Fixable Live/Dead maker	none	APC	L34994	Invitrogen
Anti-mouse CD16/32 antibody	FRC-4G8	FITC	MFCR00	Invitrogen
Anti-H2AFY antibody	EPR93592	PE-CY7	ab183041	Abcam
Murine Antibodies for Flow Cytometry				
Fixable Viability Stain 780	none	APC-cy7	565388	BD Biosciences
Anti-mouse CD3e	145-2C11	Pacific Blue	562600	BD

antibody				Biosciences
Anti-mouse CD45 antibody	30-F11	FITC	103107	Biolegend
Anti-mouse CD4 antibody	RM4-5	APC	553051	BD Biosciences
Anti-mouse CD8a antibody	53-6.7	Percp/cy5.5	100733	Biolegend
Anti-mouse CD25 antibody	PC61	Qdot 655	102038	Biolegend
Anti-mouse Nkp46 antibody	29A1.4	PE-CY7	137617	Biolegend
Anti-mouse PD-1 antibody	29F.1A12	Qdot 605	135220	Biolegend
Anti-mouse CD11b antibody	M1/70	7AAD	101228	Biolegend
Anti-mouse Ly6G antibody	RB6-8C5	PE-TexasRed	108440	Biolegend
Anti-mouse MHCII antibody	M5/114.152	AF647	107614	Biolegend
Anti-mouse CD206 antibody	C068C2	PE	141706	Biolegend
Reagents for Immunofluorescence staining				
anti-CD8	none	none	AB217344	Abcam
anti-PD-1	none	none	AB214421	Abcam
anti-IFNg	none	none	A12450	Abclonal
Other reagents				
Premium Fetal Bovine Serum	none	none	FBSSR-01021-50	Sai Ye
Serum-free Cell Cryopreservation Solution	none	none	AQ509	Aoqing
Penicillin-Streptomycin Mixed Solution	none	none	KGY0023	Keygen
Matrigengel Matrix HC	none	none	827245	ABW
Collagenase Ⅰ	none	none	C8140	Solarbio
Incomplete RPMI-1640 Medium	none	none	KGM31800N	Keygen
Red Blood Cell Lysis Buffer	none	none	CW0613S	Cwbio
PBS Buffer	none	none	KGL2206	Keygen
UltraComp eBeads™ Plus	none	none	01-3333-42	Invitrogen
DMSO	none	none	D12345	Thermo

				Fisher
Trypsin-EDTA Digest Solution	none	none	KGY0012	Keygen
Incomplete DMEM Medium	none	none	KGL1503	Keygen
dsDNase	none	none	EG20206S	Yugong Biotech
Incomplete IMDM medium	none	none	12440061	Thermo Fisher
MycoAlert kit	none	none	LT07-318	Lonza
EasySep CD14+ selection kit	none	none	17858	StemCell Technologies
LymphoPrep solution	none	none	18060	StemCell Technologies
Cell Tracer Violet (CTV) dye	none	none	C34564	Thermo Fisher
Recombinant human IFNG	none	none	300-02	PeproTech
FOXP3 buffer set	none	none	00-5523-00	Invitrogen
True-Nuclear buffer set	none	none	424401	Biolegend
Zenon labelling kit	none	none	Z25351	Invitrogen
Cell Trace Violet	none	none	C34571	Thermo Fisher Scientific

Supplementary Table .1.

The reagents used in the research institute.
