# Supplementary material for: FGFR pathway inhibition enhances PD-1 blockade and modulates antitumor immunity in colorectal cancer models
Source: BMC Cancer. 2026 Oct 13;26:1227. doi: 10.1186/s12885-026-17105-9 (PMC13670699; doi:10.1186/s12885-026-17105-9)
Supplement: Supplementary file 2 [file 12885_2026_17105_MOESM1_ESM.pdf]

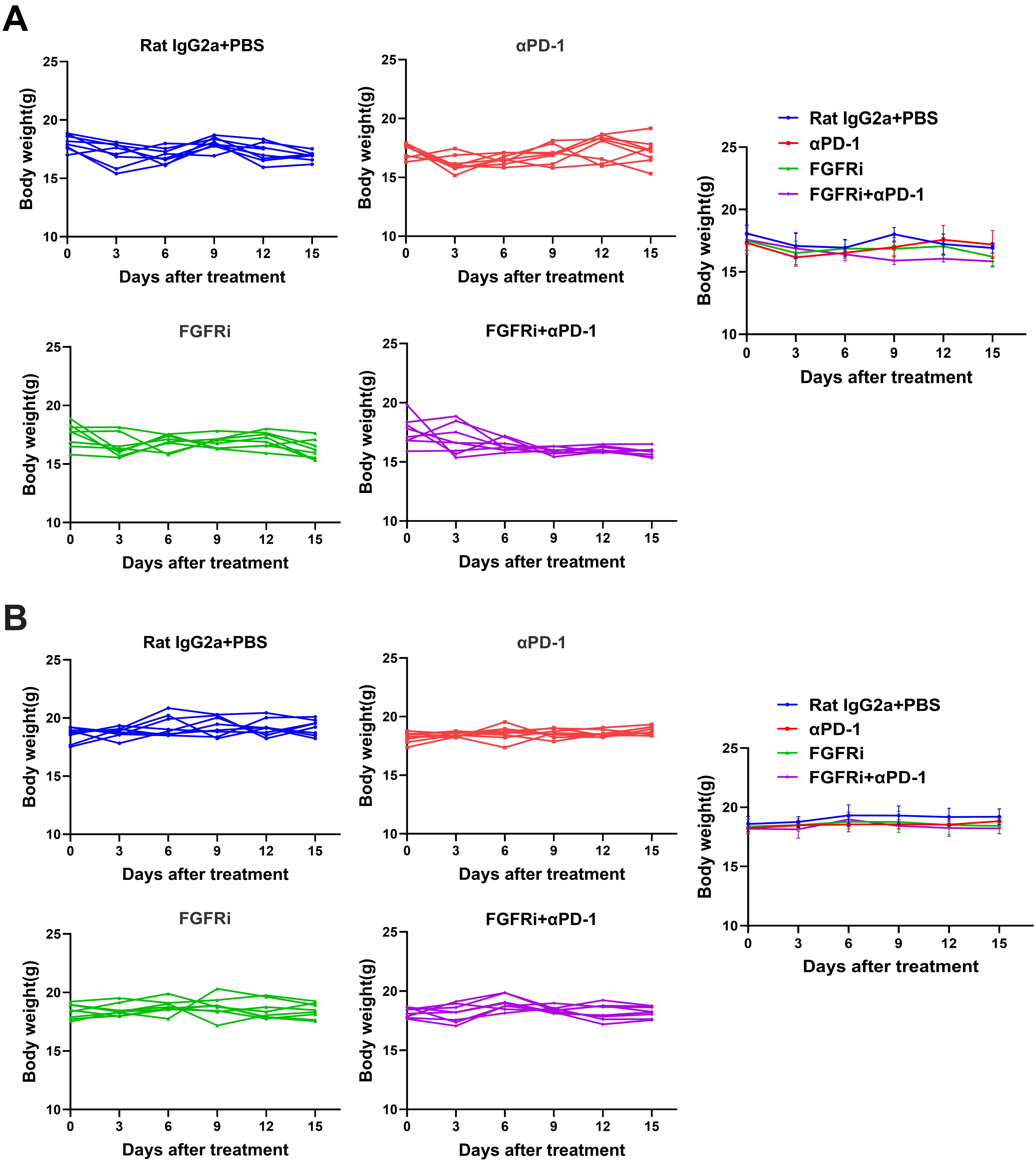
A
Rat IgG2a+PBS
αPD-1
FGFRi
FGFRi+αPD-1
Body weight(g)
Days after treatment
10
15
20
25
0
3
6
9
12
15
Rat IgG2a+PBS
αPD-1
FGFRi
FGFRi+αPD-1
B
Rat IgG2a+PBS
αPD-1
FGFRi
FGFRi+αPD-1
Body weight(g)
Days after treatment
Rat IgG2a+PBS
αPD-1
FGFRi
FGFRi+αPD-1

Supplementary Figure 1. Body weight in CT26 (MSS) and MC38 (MSI-H) mouse tumor models.

A. Individual and mean body weight measurements in CT26 tumor–bearing mice.

B. Individual and mean body weight measurements in MC38 tumor–bearing mice.
