# Supplementary material for: FGFR pathway inhibition enhances PD-1 blockade and modulates antitumor immunity in colorectal cancer models
Source: BMC Cancer. 2026 Oct 13;26:1227. doi: 10.1186/s12885-026-17105-9 (PMC13670699; doi:10.1186/s12885-026-17105-9)
Supplement: Supplementary file 3 [file 12885_2026_17105_MOESM2_ESM.pdf]

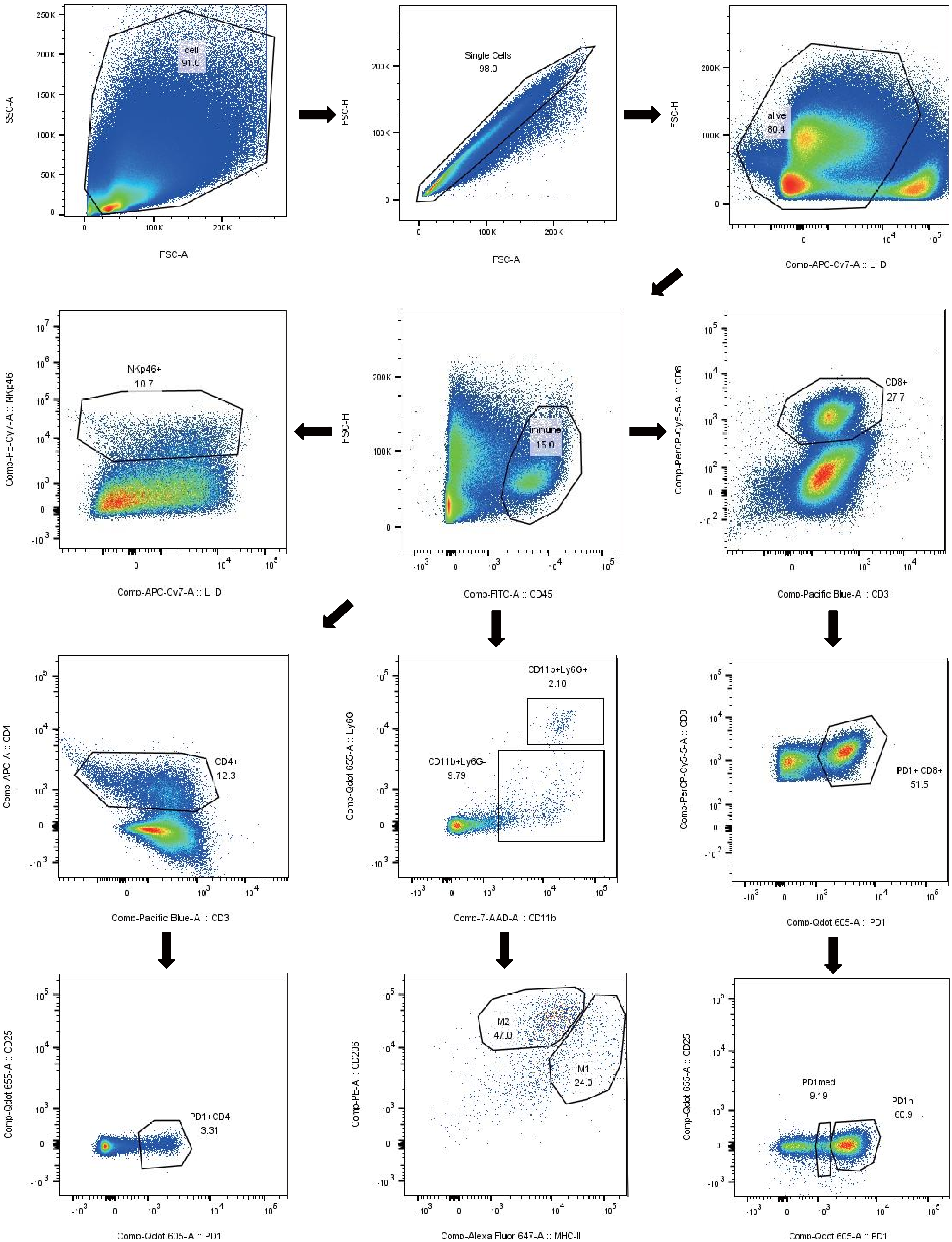
cell
91.0
SSC-A
FSC-A
Single Cells
98.0
FSC-H
alive
80.4
Comp-APC-Cy7-A :: L_D
NKp46+
10.7
Comp-PE-Cy7-A :: NKp46
Immune
15.0
Comp-FITC-A :: CD45
CD8+
27.7
Comp-PerCP-Cy5-5-A :: CD8
Comp-Pacific Blue-A :: CD3
CD4+
12.3
Comp-APC-A :: CD4
CD11b+Ly6G+
2.10
CD11b+Ly6G-
9.79
Comp-Qdot 655-A :: Ly6G
Comp-7-AAD-A :: CD11b
PD1+ CD8+
51.5
Comp-Qdot 605-A :: PD1
PD1+CD4
3.31
Comp-Qdot 655-A :: CD25
M2
47.0
M1
24.0
Comp-PE-A :: CD206
Comp-Alexa Fluor 647-A :: MHC-II
PD1med
9.19
PD1hi
60.9

Supplementary Figure 2. Gating strategy for flow cytometry analysis of immune cell subsets in CRC tumors.

$CD4^+$ T cells, $CD8^+$ T cells, NK cells, $PD1^+$ $CD4^+$ T cells, $PD1^+$ $CD8^+$ T cells, $PD1^{hi}$ $CD8^+$ T cells, M1 TAM and M2 TAM were gated as shown in the figure. (M1 TAM: M1 tumor-associated macrophages, M2 TAM: M2 tumor-associated macrophages.)
