# Supplementary material for: FGFR pathway inhibition enhances PD-1 blockade and modulates antitumor immunity in colorectal cancer models
Source: BMC Cancer. 2026 Oct 13;26:1227. doi: 10.1186/s12885-026-17105-9 (PMC13670699; doi:10.1186/s12885-026-17105-9)
Supplement: Supplementary file 4 [file 12885_2026_17105_MOESM3_ESM.pdf]

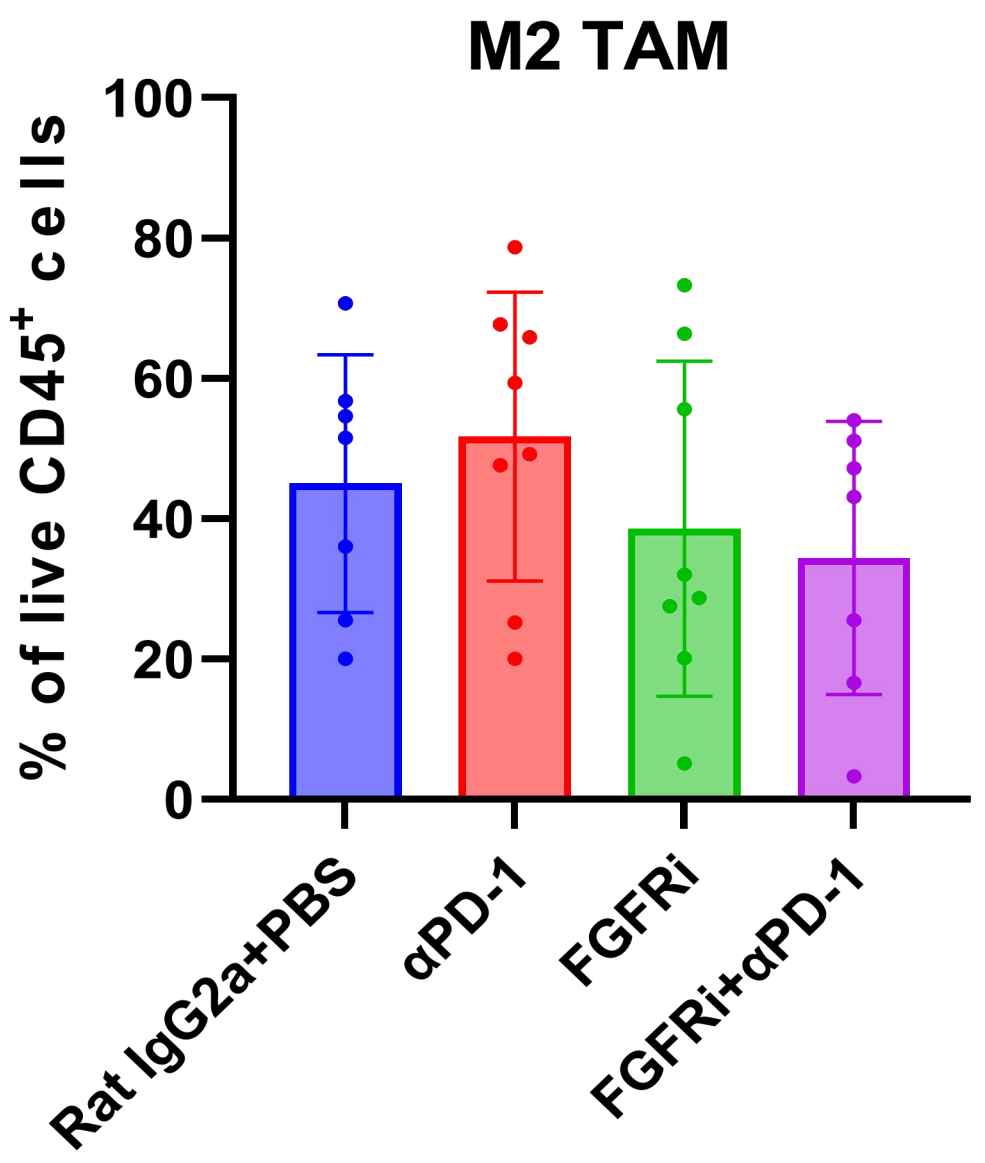

Supplementary Figure 3.
Percentage of M2 tumor-associated macrophages (M2 TAM) in CD45⁺ TILs. (one-way ANOVA with Tukey's correction, n=8.)
