# Supplementary material for: FGFR pathway inhibition enhances PD-1 blockade and modulates antitumor immunity in colorectal cancer models
Source: BMC Cancer. 2026 Oct 13;26:1227. doi: 10.1186/s12885-026-17105-9 (PMC13670699; doi:10.1186/s12885-026-17105-9)
Supplement: Supplementary file 5 [file 12885_2026_17105_MOESM4_ESM.pdf]

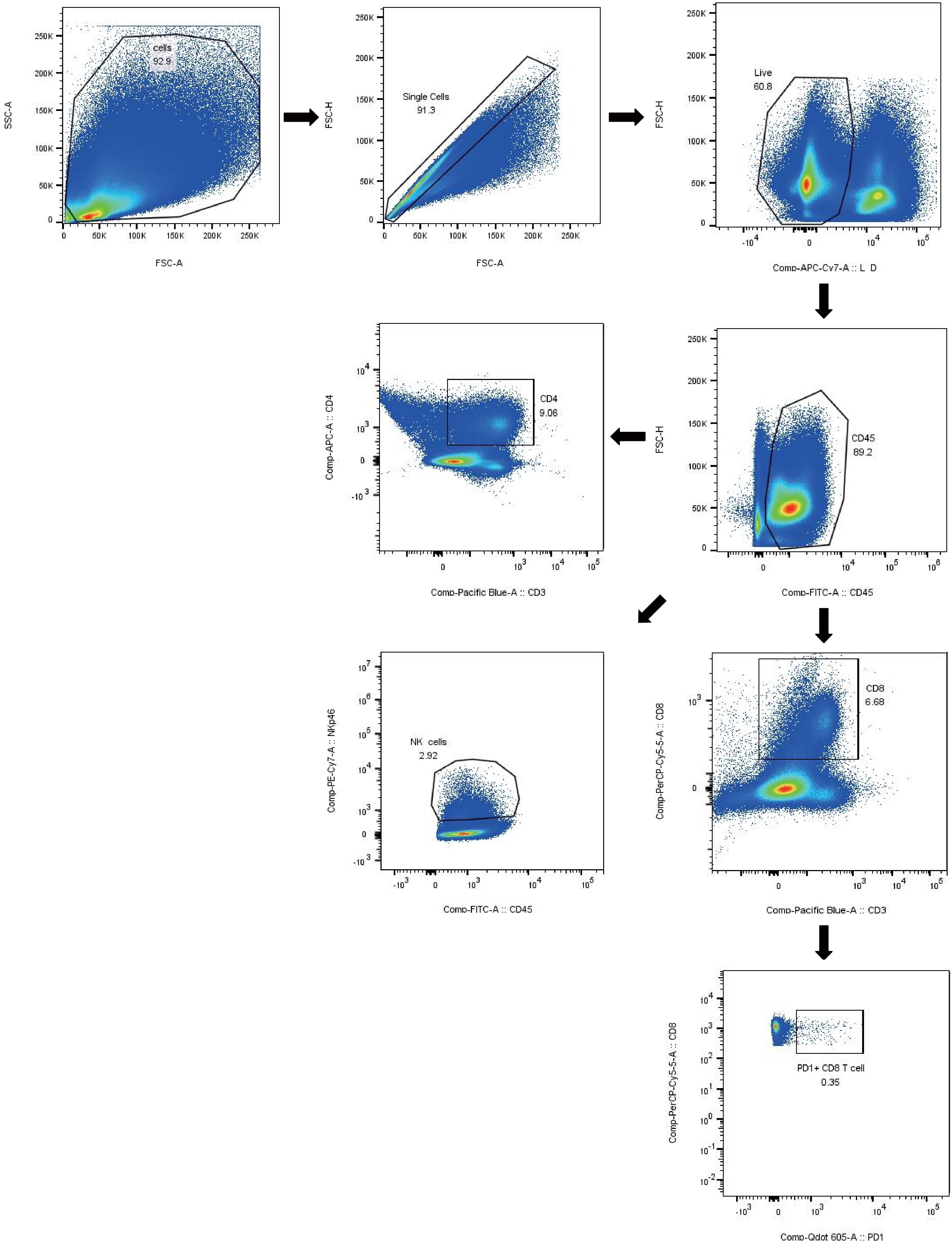

cells
92.9
SSC-A
FSC-A
Single Cells
91.3
FSC-H
FSC-A
Live
60.8
FSC-H
Comp-APC-Cy7-A :: L_D
CD45
89.2
FSC-H
Comp-FITC-A :: CD45
CD4
9.06
Comp-APC-A :: CD4
Comp-Pacific Blue-A :: CD3
CD8
6.68
Comp-PerCP-Cy5-5-A :: CD8
Comp-Pacific Blue-A :: CD3
NK cells
2.92
Comp-PE-Cy7-A :: NKp46
Comp-FITC-A :: CD45
PD1+ CD8 T cell
0.35
Comp-PerCP-Cy5-5-A :: CD8
Comp-Qdot 605-A :: PD1

Supplementary Figure 4. Gating strategy for flow cytometry analysis of spleen.

$CD4^+$ T cells, $CD8^+$ T cells, NK cells and $PD1^+$ $CD8^+$ T cells were gated as shown in the figure.
