# Supplementary material for: FGFR pathway inhibition enhances PD-1 blockade and modulates antitumor immunity in colorectal cancer models
Source: BMC Cancer. 2026 Oct 13;26:1227. doi: 10.1186/s12885-026-17105-9 (PMC13670699; doi:10.1186/s12885-026-17105-9)
Supplement: Supplementary file 6 [file 12885_2026_17105_MOESM5_ESM.pdf]

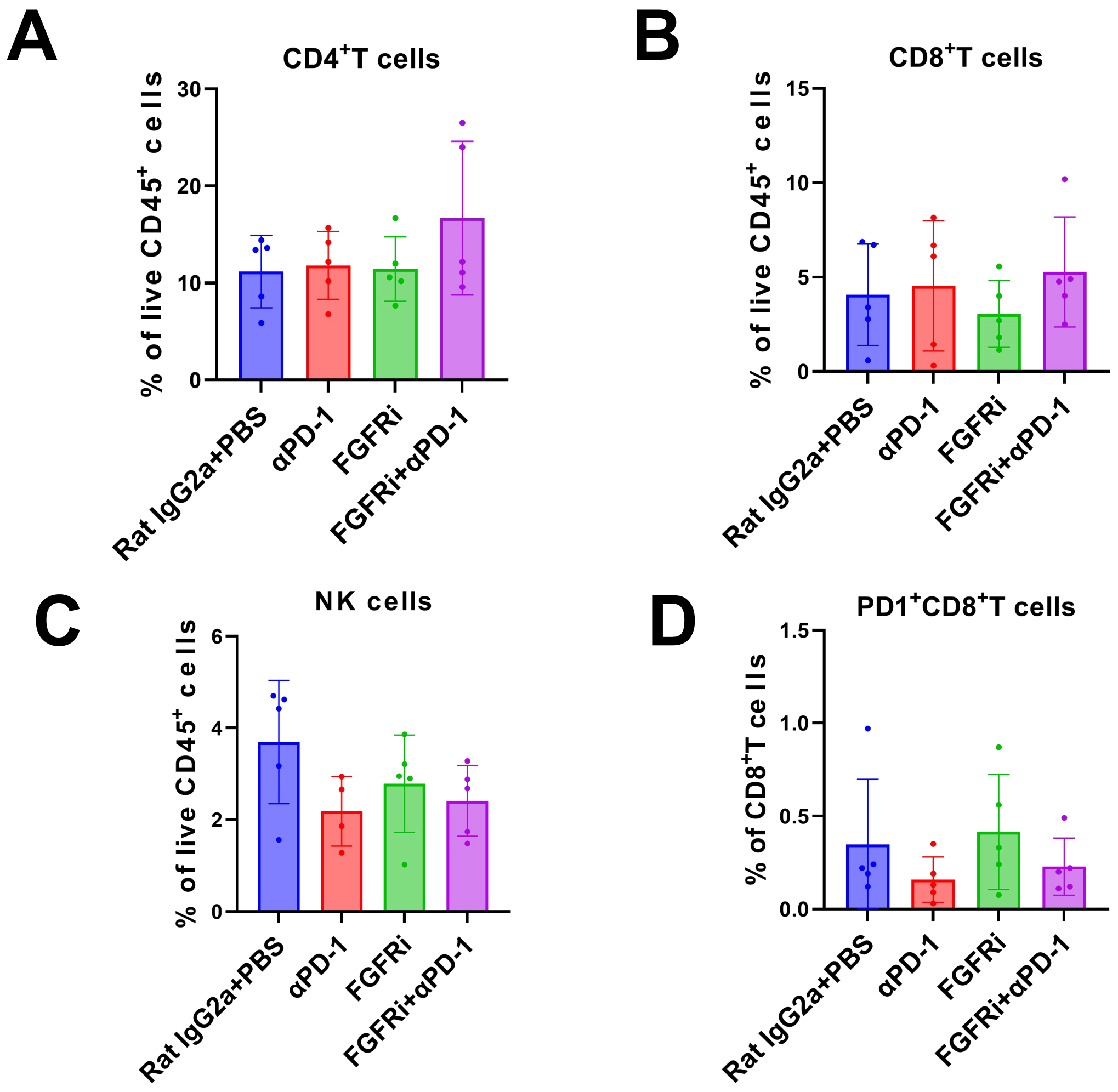

Supplementary Figure 5. Immune modulatory effects of anti–PD-1, FGFRi, and their combination in the spleen.
A–D. The percentages of $CD4^+$ T cells, $CD8^+$ T cells, NK cells within $CD45^+$ splenocytes, and the percentage of $PD\text{-}1^+$ $CD8^+$ T cells within total $CD8^+$ T cells are shown.
