# Supplementary material for: FGFR pathway inhibition enhances PD-1 blockade and modulates antitumor immunity in colorectal cancer models
Source: BMC Cancer. 2026 Oct 13;26:1227. doi: 10.1186/s12885-026-17105-9 (PMC13670699; doi:10.1186/s12885-026-17105-9)
Supplement: Supplementary file 7 [file 12885_2026_17105_MOESM6_ESM.pdf]

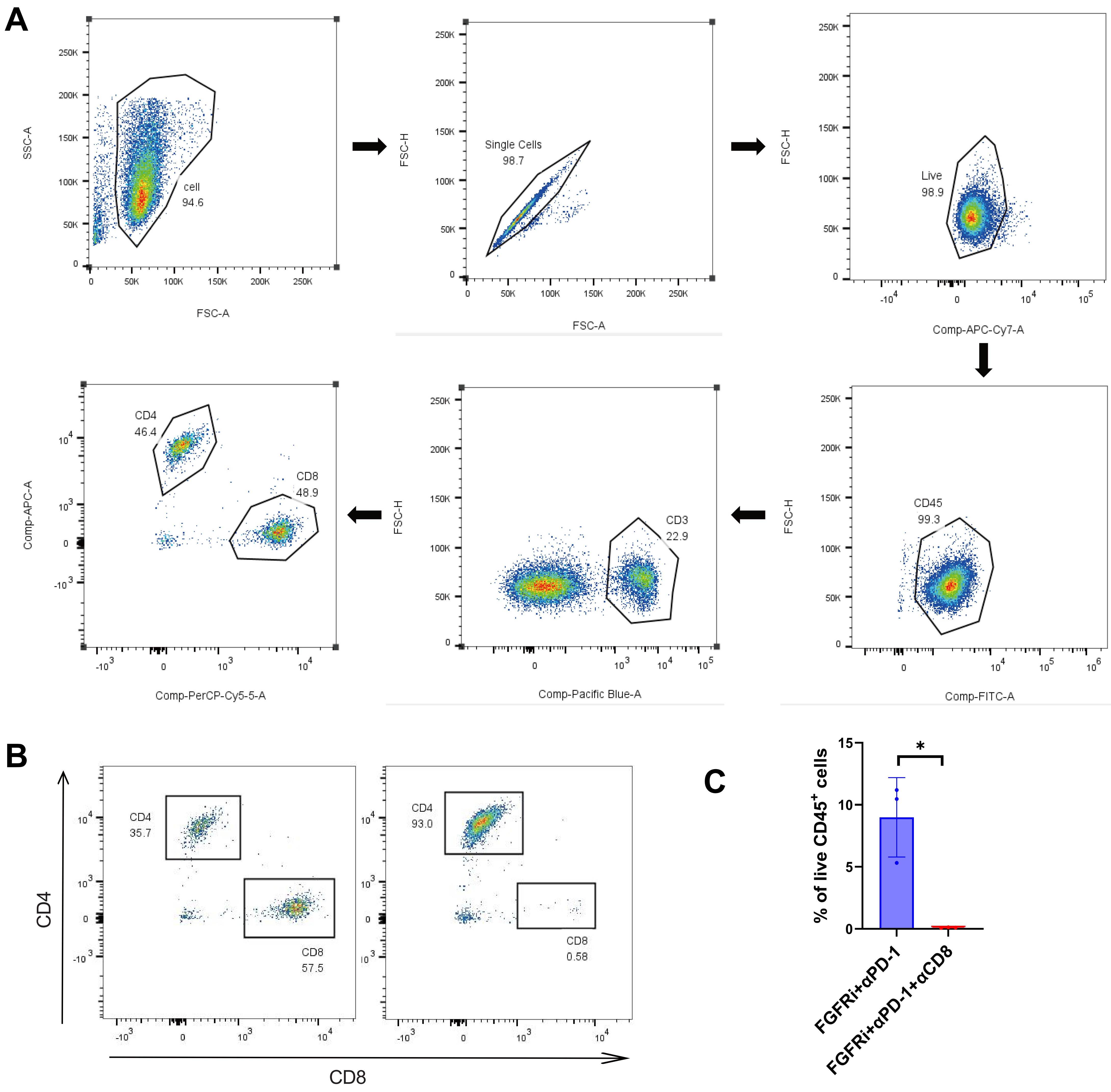

A
SSC-A
FSC-A
cell
94.6
FSC-H
Single Cells
98.7
Live
98.9
Comp-APC-Cy7-A
CD45
99.3
Comp-FITC-A
CD3
22.9
Comp-Pacific Blue-A
CD4
46.4
CD8
48.9
Comp-APC-A
Comp-PerCP-Cy5-5-A
B
CD4
35.7
CD8
57.5
CD4
93.0
CD8
0.58
CD4
CD8
C
% of live CD45+ cells
*
FGFRi+αPD-1
FGFRi+αPD-1+αCD8

Supplementary Figure 6. Verification of complete CD8$^+$ T cell depletion.

A. Gating strategy for identifying CD8$^+$ and CD4$^+$ T cells by flow cytometry. **B, C.** The proportion of CD8$^+$ T cells and CD4$^+$ T cells to CD45$^+$ cells in the FGFRi+anti-PD-1 group and FGFRi+anti-PD-1+anti-CD8 depletion group.
