# Supplementary material for: FGFR pathway inhibition enhances PD-1 blockade and modulates antitumor immunity in colorectal cancer models
Source: BMC Cancer. 2026 Oct 13;26:1227. doi: 10.1186/s12885-026-17105-9 (PMC13670699; doi:10.1186/s12885-026-17105-9)
Supplement: Supplementary file 8 [file 12885_2026_17105_MOESM7_ESM.pdf]

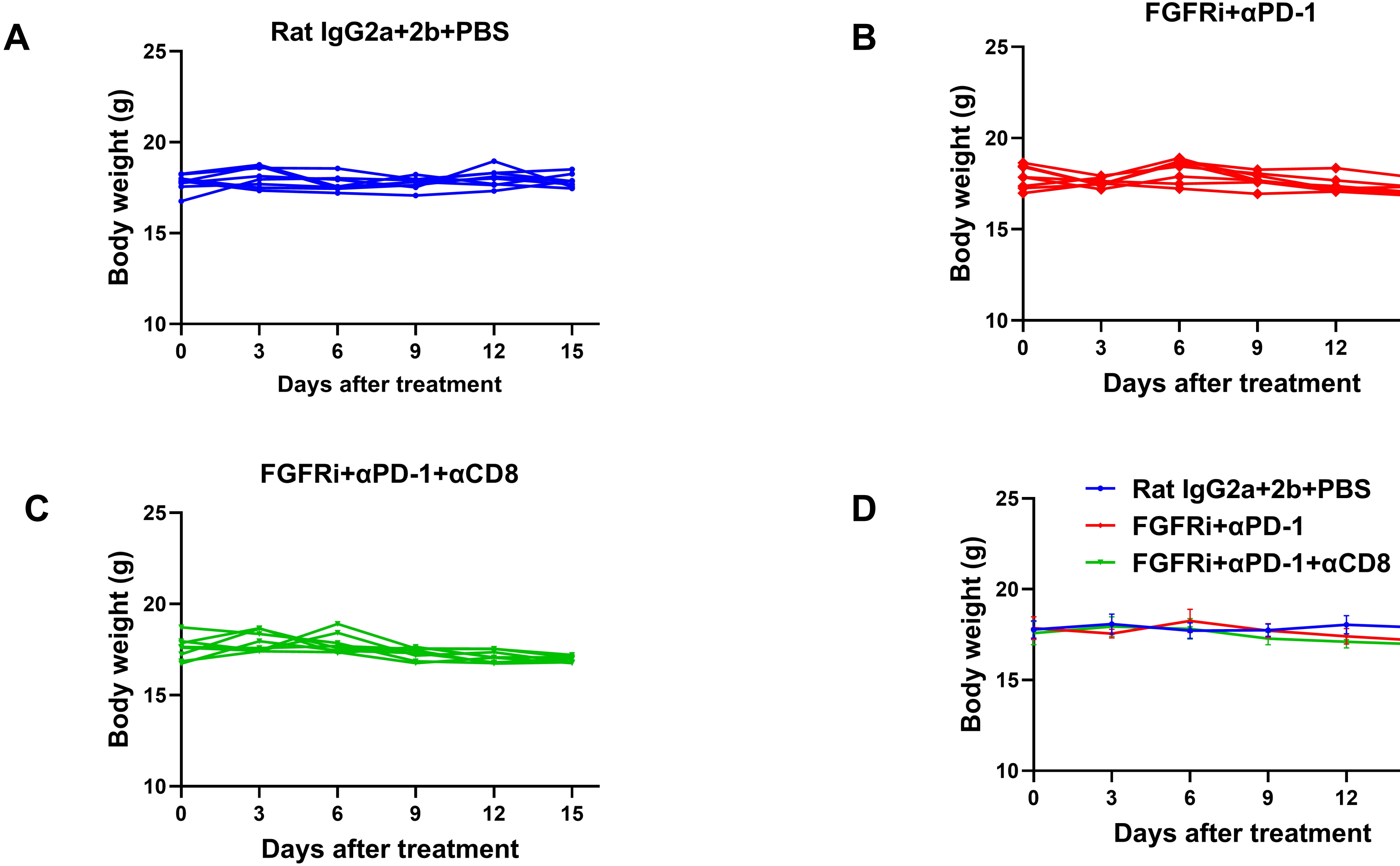

A
Rat IgG2a+2b+PBS
Body weight (g)
Days after treatment
B
FGFRi+αPD-1
Body weight (g)
Days after treatment
C
FGFRi+αPD-1+αCD8
Body weight (g)
Days after treatment
D
Rat IgG2a+2b+PBS
FGFRi+αPD-1
FGFRi+αPD-1+αCD8
Body weight (g)
Days after treatment

Supplementary Figure 7. Body weight changes in CT26 mouse tumor models.
A. Individual body weight of mice in Rat IgG2a+2b+PBS treatment group. **B.** Individual body weight of mice in FGFRi+anti-PD-1 treatment group. **C.** Individual body weight of mice in FGFRi+anti-PD-1+anti-CD8 treatment group. **D.** Mean body weight in each treatment group.
