# Supplementary material for: FGFR pathway inhibition enhances PD-1 blockade and modulates antitumor immunity in colorectal cancer models
Source: BMC Cancer. 2026 Oct 13;26:1227. doi: 10.1186/s12885-026-17105-9 (PMC13670699; doi:10.1186/s12885-026-17105-9)
Supplement: Supplementary file 9 [file 12885_2026_17105_MOESM8_ESM.pdf]

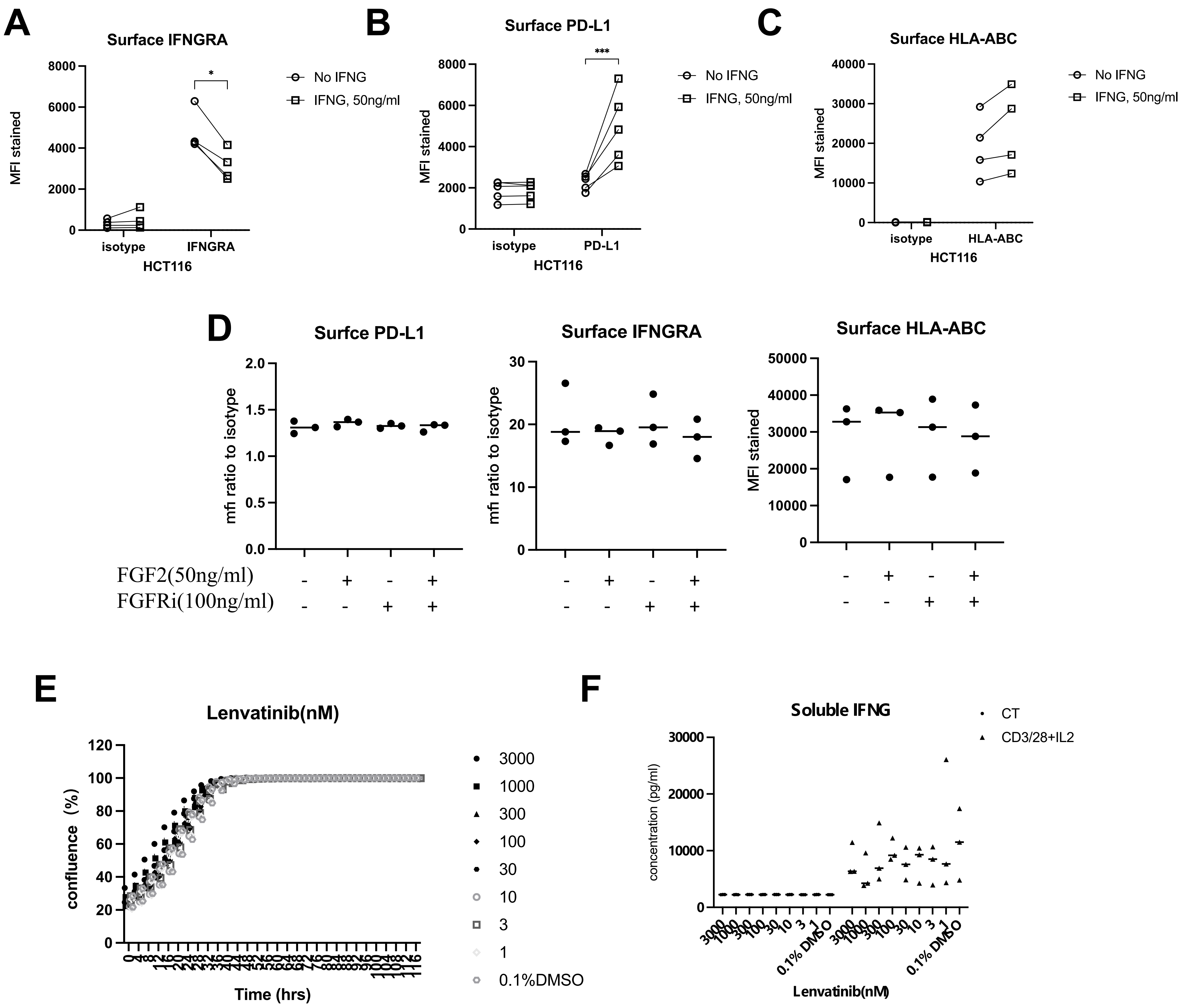
A
Surface IFNGRA
MFI stained
isotype
IFNGRA
HCT116
No IFNG
IFNG, 50ng/ml
B
Surface PD-L1
MFI stained
isotype
PD-L1
HCT116
No IFNG
IFNG, 50ng/ml
C
Surface HLA-ABC
MFI stained
isotype
HLA-ABC
HCT116
No IFNG
IFNG, 50ng/ml
D
Surfce PD-L1
mfi ratio to isotype
Surface IFNGRA
mfi ratio to isotype
Surface HLA-ABC
MFI stained
FGF2(50ng/ml)
FGFRi(100ng/ml)
E
Lenvatinib(nM)
confluence (%)
Time (hrs)
3000
1000
300
100
30
10
3
1
0.1%DMSO
F
Soluble IFNG
concentration (pg/ml)
Lenvatinib(nM)
CT
CD3/28+IL2
0.1% DMSO

Supplementary Figure 8. The effects of IFNg and lenvatinib treatment on HCT116 cells.

A. IFNg treatment significantly upregulated PD-L1 expression in HCT116 cells. **B.** IFNg treatment had no substantial effect on HLA-ABC expression in HCT116 cells. **C.** IFNg treatment significantly downregulated IFNGR1 expression in HCT116 cells. **D.** Treatment with FGF2 and lenvatinib did not affect the expression of PD-L1, IFNGR1, or HLA-ABC in HCT116 cells. **E.** Lenvatinib treatment did not affect HCT116 cell proliferation. **F.** Lenvatinib treatment did not affect the activation of primary human lymphocytes. (*， $p < 0.05$, ***， $p < 0.001$ vs. isotype (t tests or nonparametric tests).
