# Supplementary material for: FGFR pathway inhibition enhances PD-1 blockade and modulates antitumor immunity in colorectal cancer models
Source: BMC Cancer. 2026 Oct 13;26:1227. doi: 10.1186/s12885-026-17105-9 (PMC13670699; doi:10.1186/s12885-026-17105-9)
Supplement: Supplementary file 10 [file 12885_2026_17105_MOESM9_ESM.pdf]

NOTE: Please save this file locally before filling in the table, DO NOT work on the file within your internet browser as changes will not be saved. Adobe Acrobat Reader (available free here) is recommended for completion.

The ARRIVE guidelines 2.0: author checklist

The ARRIVE Essential 10

These items are the basic minimum to include in a manuscript. Without this information, readers and reviewers cannot assess the reliability of the findings.

Item		Recommendation	Section/line number, or reason for not reporting
**Study design**	1	For each experiment, provide brief details of study design including:<br>a. The groups being compared, including control groups. If no control group has been used, the rationale should be stated.<br>b. The experimental unit (e.g. a single animal, litter, or cage of animals).	The study used syngeneic mouse models of colorectal cancer to evaluate the antitumor efficacy of FGFR inhibition in combination with PD-1 immune checkpoint blockade. Four treatment arms were included: vehicle control, FGFR inhibitor alone, anti-PD-1 antibody alone, and combination therapy.<br>The experimental unit was a cage of animals.
**Sample size**	2	a. Specify the exact number of experimental units allocated to each group, and the total number in each experiment. Also indicate the total number of animals used.<br>b. Explain how the sample size was decided. Provide details of any *a priori* sample size calculation, if done.	MC38 mouse models, for a total of 64 mice. For the flow cytometry experiment, there were 8 mice in each group, for a total of 32 mice. In the tumor re-challenge experiment, the control group consisted of 5 mice, while<br>b.The sample size was determined based on prior literature and effect size estimates. No formal power calculation was conducted; however, the group size aligns with that of similar immunotherapy studies.
**Inclusion and exclusion criteria**	3	a. Describe any criteria used for including and excluding animals (or experimental units) during the experiment, and data points during the analysis. Specify if these criteria were established *a priori*. If no criteria were set, state this explicitly.<br>b. For each experimental group, report any animals, experimental units or data points not included in the analysis and explain why. If there were no exclusions, state so.<br>c. For each analysis, report the exact value of *n* in each experimental group.	weeks old, healthy, and weighed between 18~ 22 g at baseline. Animals were excluded from analysis if they showed signs of illness unrelated to tumor progression, failed to recover from tumor implantation, or died<br>b.In the flow cytometry experiment, two mice were excluded due to poor hatching color of antibodies, and in the RNAseq experiment, one mouse was excluded due to significant differences in gene expression<br>group consisted of eight mice. In the flow cytometry experiment, six mice were analyzed for M1 TAM results, and eight mice were analyzed for the remaining results. In the RNAseq experiment, the PD-1 monotherapy group consisted of seven mice
**Randomisation**	4	a. State whether randomisation was used to allocate experimental units to control and treatment groups. If done, provide the method used to generate the randomisation sequence.<br>b. Describe the strategy used to minimise potential confounders such as the order of treatments and measurements, or animal/cage location. If confounders were not controlled, state this explicitly.	treatment or control groups using computer-generated randomization sequences. Randomization was performed when tumor volumes in the CT26 mouse model reached approximately 30~ 60 mm³ and again when volumes reached 70~ 110 mm³, to ensure comparable tumor burden<br>animals from all treatment groups were housed under identical environmental conditions (temperature, humidity, light/dark cycle) and randomly distributed across cages and cage positions on the rack. Treatment and sample collection were performed at the same time of day for all
**Blinding**	5	Describe who was aware of the group allocation at the different stages of the experiment (during the allocation, the conduct of the experiment, the outcome assessment, and the data analysis).	investigator not involved in treatment administration or data analysis. The personnel responsible for treatment administration were aware of group assignments, but outcome assessment (e.g., tumor measurement, flow cytometry analysis) and data analysis were conducted by investigators blinded to treatment groups
**Outcome measures**	6	a. Clearly define all outcome measures assessed (e.g. cell death, molecular markers, or behavioural changes).<br>b. For hypothesis-testing studies, specify the primary outcome measure, i.e. the outcome measure that was used to determine the sample size.	lymphocytes was measured by flow cytometry. Macrophage polarization: M1 and M2 macrophages in tumors were quantified by flow cytometry.<br>The main outcome measure of hypothesis testing is the change in tumor volume after treatment in each group, while secondary outcome measures include immune cell infiltration (CD8 T cells, PD-1 T cells), cytokine secretion, and mouse survival time
**Statistical methods**	7	a. Provide details of the statistical methods used for each analysis, including software used.<br>b. Describe any methods used to assess whether the data met the assumptions of the statistical approach, and what was done if the assumptions were not met.	Tukey Ľ s multiple comparisons test. Survival curves were evaluated using the Kaplan~ Meier method, with statistical significance determined by the log-rank test. Transcriptomic data (RNA-seq) were<br>Data were assessed for normality using the Shapiro~ Wilk test and for homogeneity of variances using Levene Ľ s test where applicable. A p-value of <0.05 was considered statistically significant
**Experimental animals**	8	a. Provide species-appropriate details of the animals used, including species, strain and substrain, sex, age or developmental stage, and, if relevant, weight.<br>b. Provide further relevant information on the provenance of animals, health/immune status, genetic modification status, genotype, and any previous procedures.	CT26 mouse model (BALB/c mice) MC38 mouse model (C57BL/6 mice) Gender: Female Age/developmental stage: 8 weeks old Weigh 18-22g<br>Raised in SPF grade animal facilities (temperature 22 ± 2 ° C, humidity 50 ± 10%, 12/12 hour light dark cycle) Health status: Healthy Genetic modification: wild-type
**Experimental procedures**	9	For each experimental group, including controls, describe the procedures in enough detail to allow others to replicate them, including:<br>a. What was done, how it was done and what was used.<br>b. When and how often.<br>c. Where (including detail of any acclimatisation periods).<br>d. Why (provide rationale for procedures).	4. All sampling operations were conducted under SPF conditions, and the operators were trained and qualified to use sterile tools. 5. Tumor tissue analysis is used to evaluate the effects of drugs on immune cell subsets<br>200 μg (10 mg/kg) anti-mouse PD-1 antibody (Bio X Cell, BE0146) or isotype control IgG (Bio X Cell) on days 0, 3, 6, and 9. FGFRi (10 mg/kg) was administered orally daily from days 0 to 14. Mouse weight and tumor<br>central laboratory of Shandong First Medical University, strictly following the aseptic procedures during the experiment. All mice were adaptively fed for one week before the<br>previous research Combination therapy interval: avoiding pharmacokinetic interactions Tumor termination criteria: volume>2000 mm
**Results**	10	For each experiment conducted, including independent replications, report:<br>a. Summary/descriptive statistics for each experimental group, with a measure of variability where applicable (e.g. mean and SD, or median and range).<br>b. If applicable, the effect size with a confidence interval.	3. IHC result score: calculated as the average number of positive cells ± SD per image; 4. In transcriptome data analysis, expression levels are expressed as TPM or FPKM, and differential expression is determined using<br>After conducting analysis of variance (ANOVA), Tukey's test was used to compare multiple groups, and a p-value less than 0.05 was considered statistically significant.

The Recommended Set

These items complement the Essential 10 and add important context to the study. Reporting the items in both sets represents best practice.

Item		Recommendation	Section/line number, or reason for not reporting
**Abstract**	11	Provide an accurate summary of the research objectives, animal species, strain and sex, key methods, principal findings, and study conclusions.	We investigated the immunomodulatory effects
**Background**	12	a. Include sufficient scientific background to understand the rationale and context for the study, and explain the experimental approach.<br>b. Explain how the animal species and model used address the scientific objectives and, where appropriate, the relevance to human biology.	fibroblast growth factor receptor (FGFR) signaling pathway contributes to tumor immune evasion, yet its role in CRC remains insufficiently understood.The necessity of using FGFR inhibitors in combination with PD1 monoclonal antibody has been<br>This study used two colorectal cancer models with immune active mouse strains (BALB/c and C57BL/6) to simulate the microsatellite stable (MSS) and highly microsatellite unstable (MSI-H) subtypes of human CRC, covering common clinical molecular features.
**Objectives**	13	Clearly describe the research question, research objectives and, where appropriate, specific hypotheses being tested.	Although PD-1 inhibitors have shown efficacy i
**Ethical statement**	14	Provide the name of the ethical review committee or equivalent that has approved the use of animals in this study, and any relevant licence or protocol numbers (if applicable). If ethical approval was not sought or granted, provide a justification.	This study was conducted in compliance with all applicable ethical regulations and was approved by the Review Committee of Shandong First Medical University Affiliated Provincial Hospital (Shandong Provincial Hospital) (Approval No.: No.2023-065). All authors are informed and agree to participate in this study.
**Housing and husbandry**	15	Provide details of housing and husbandry conditions, including any environmental enrichment.	Facility level: SPF (pathogen free) animal room
**Animal care and monitoring**	16	a. Describe any interventions or steps taken in the experimental protocols to reduce pain, suffering and distress.<br>b. Report any expected or unexpected adverse events.<br>c. Describe the humane endpoints established for the study, the signs that were monitored and the frequency of monitoring. If the study did not have humane endpoints, state this.	a.Isoflurane inhalation anesthesia is used during tumor inoculation (induction 5%, maintenance 1.5-2%). In addition, animals in the same cage are treated in the same group to avoid social isolation<br>b.The mice in the combination therapy group showed mild weight loss, but did not meet the criteria for euthanasia<br>endpoints were reached, including tumor ulceration or a 30% body weight loss compared to day 0. Survival duration (in days) was defined as the time from day 0 until euthanasia or death. Survival curves for
**Interpretation/ scientific implications**	17	a. Interpret the results, taking into account the study objectives and hypotheses, current theory and other relevant studies in the literature.<br>b. Comment on the study limitations including potential sources of bias, limitations of the animal model, and imprecision associated with the results.	that FGFR pathway activation contributes to immune evasion mechanisms in various tumor types. Our findings suggest that FGFR inhibition may reverse immune suppression and increase T-cell infiltration, thereby improving response to PD-1 blockade.<br>spontaneous colorectal cancers in humans. This limits the generalizability of the findings to clinical settings. Additionally, the immune response observed in syngeneic mouse models may differ from that in human patients due to species-specific differences in immune
**Generalisability/ translation**	18	Comment on whether, and how, the findings of this study are likely to generalise to other species or experimental conditions, including any relevance to human biology (where appropriate).	antibodies drive immune activation, providing a mechanistic framework for the combined immunotherapy of MSS type CRC. The conversion needs to be promoted through the following steps:<br>Validate safety in non-human primate models; Conduct phase II trials targeting patients with high FGFR3 expression and
**Protocol registration**	19	Provide a statement indicating whether a protocol (including the research question, key design features, and analysis plan) was prepared before the study, and if and where this protocol was registered.	19.All research questions, key design features, and analysis plans of this study were developed in detail before the start of the study. In addition, the ethics of mouse experiments have been applied for before the start of the experiment
**Data access**	20	Provide a statement describing if and where study data are available.	20.RNA sequencing data У generated in this st
**Declaration of interests**	21	a. Declare any potential conflicts of interest, including financial and non-financial. If none exist, this should be stated.<br>b. List all funding sources (including grant identifier) and the role of the funder(s) in the design, analysis and reporting of the study.	The authors declare that they have no competing interests.<br>of Jinan City (202134027). Y.M. L̓ s research group is generously supported by grants from the SciLifeLab (SLL2019/9, SciLifeLab Fellowship), the Swedish Foundation for Strategic Research (FFL-0043), and the
